# Major chemical constituents from *Illicium griffithii* Hook. f. & Thoms of North East India and their cytotoxicity and antimicrobial activities

**DOI:** 10.55730/1300-0527.3451

**Published:** 2022-04-08

**Authors:** Deepjyoti DUTTA, Bardwi NARZARY, Snigdha SAIKIA, Kashyap Jyoti TAMULI, Manobjyoti BORDOLOI, Neipihoi LHOUVUM, Shyamalendu NATH, Ranjan Kumar SAHOO, Barnali GOGOI, Surovi SAIKIA, Nayan Kamal BHATTACHARYYA

**Affiliations:** 1 Chemical Science and Technology Division, CSIR-North East Institute of Science and Technology, Jorhat, Assam, India; 2 Department of Chemistry, Cotton University, Guwahati, Assam, India; 3 Department of Chemistry, Sikkim Manipal University, Majitar, Sikkim, India

**Keywords:** *Illicium griffithii*, North East India, prenylated allyl derivatives, antimicrobial activity, cytotoxicity

## Abstract

*Illicium griffithii* Hook. f. & Thoms is an endemic medicinal plant of North East India found in the Eastern Himalayan region of biodiversity mega centre. Herein, chemical investigation of *I. griffithii*, afforded five compounds and their structures were determined through extensive use of NMR, HRMS, and FT-IR spectroscopy. The complete proton-proton, proton-carbon coupling network of compound **1** was determined using ^1^H-^1^H COSY, HSQC and NOESY NMR experiments. All the compounds were evaluated for their cytotoxic activity by MTT assay and antimicrobial activity by Agar well diffusion method. Compound **1** exhibited significant cytotoxicity activity against Lung cancer (A549) and pancreatic cancer (MIAPaCa2) cell lines with IC_50_ values of 15.01 ± 2.69 μg/mL and 47.77 ± 2.38 μg/mL, respectively. Further, the compound **1** exhibited good antimicrobial activities against *Escherichia coli* and *Candida albicans* with MIC 7.50 ± 0.28 μg/mL and 7.50 ± 0.86 μg/mL, respectively. The other isolated compounds along with the extracts of *I. griffithii* also displayed moderate anticancer and antimicrobial activities against respective strains. To the best of our knowledge, this is the first study of isolation of compounds from bark, wood, and leaf along with cytotoxicity and antimicrobial activities of *I. griffithii* from the North Eastern region of India and could be a potential herbal medicine in near future.

## 1. Introduction

North East India is rich in many ethno-botanical plants with tremendous traditional uses. Among such medicinal plants, *Illicium griffithii* is an endemic plant of North East India found in the eastern Himalayan region at an altitude of 1400–1700 m in subtropical and temperate forests [1,2]. Its fruit has a star-like shape with a shining boat-shaped seed pod and has a slightly aromatic, bitter with astringent taste [3]. The genus *Illicium* belongs to the family Illiciaceae and has numerous sources of chemical constituents, such as, prenylated C_6_-C_3_ compounds, sesquiterpenoids, flavonoids, glycosides, lignans and phenylpropanoids etc. [4]. Many of them have several biological activities including enhanced choline acetyltransferase, anticancer, antiviral, antiinflammatory, and anti-HIV activities [5–8]. A literature survey depicts that, the fruit of *I. griffithii* has been shown to have anticancer [9], antimicrobial [10], and antioxidant [11] activities. In our earlier study, we have identified the fruit of *I. griffithii* as a rich source of Shikimic acid which is a raw material for the production of oseltavir (Tamiflu), the only drug available for swine flu and avian flu [1].

The previous study on this plant has proved that it has some major compounds like neoanisatin, anisotin, nisatin, and pseudoanisatin etc. [12]. Moreover, various components of essential oil have been identified from the different parts of *I. griffithii*, for which it has high demand in the spice and perfumery industries [13,14]. In addition to that, a recent study displayed that, a new neolignan from *Illicium difengpi* [15], allo-cedrane sesquiterpene glycoside from *Illicium Simonsii* [16], and illifargeins A–M with one norillifargeal A from *Illicium fargesii* [17] has been isolated and showed potential activities against Coxsackievirus B3 and antiinflammatory activity respectively. Hence, further studies need to be done comprehensively to understand its potential for potential drug discovery to improve our knowledge and appreciation. As part of our ongoing research in search of bioactive plant derived compounds from the plants of North East India [18, 19, 20], we have investigated the leaves, bark, and wood of this plant and the results are presented herein. However, there is no report available till now to identify these bioactive compounds with proper spectroscopic analyses from the parts of *I. griffithii*.

## 2. Materials and methods

### 2.1. General

Fractionation and isolation of compounds were done by column chromatography using 60–120 mesh silica gels. TLC experiments were carried out using precoated Silica gel 60 F_254_ sheets (Merck, Darmstadt, Germany). 1D and 2D NMR were recorded with a Bruker AVANCE DPX 500 MHz NMR spectrometer, Switzerland with tetramethylsilane (TMS) as the internal reference. Melting points were measured using BUCHI M-560 capillary melting point apparatus. High-Resolution Electro-Spray Ionization Mass spectra (HR-ESI-MS) were recorded using Waters XEVO G2-Xs QT LC-MS system. FT-IR spectra were recorded in Elmer FT-IR 2000 spectrometer on a thin film using chloroform. A549 Lung cancer and MIAPaCa2 pancreatic cancer cell lines were purchased from NCCS, Pune, India. MTT was purchased from Sigma-Aldrich Co (St Louis, MO, USA). The absorbance was measured on an ELISA plate reader (FilterMax F3 Multi-Mode Microplate Readers, Molecular Devices) with a test wavelength of 570 nm and a reference wavelength of 630 nm.

### 2.2. Plant material

The leaves, bark, and wood of *I. griffithii* were collected by Prof. M. Bordoloi from Arunachal Pradesh, India in July 2016. The plant material was identified by Prof. M. Bordoloi and a voucher specimen (No. NPC/298-300) was deposited at the CSIR-North East Institute of Science and Technology, Jorhat, India.

### 2.3. Extraction and isolation

The dried powder of *I. griffithii* barks (1 kg), woods (1 kg), and leaves (200 g) were extracted with ethanol and concentrated under vacuum at 45 °C to obtain three extracts barks (IGB), wood (IGW), and leaves (IGL). The yield of the ethanol extracts obtained corresponds to 136 g (yield 13.60%), 113 g (yield 11.30%), and 10 g (yield 5%) for barks, wood and leaves, respectively. The extracts were vacuum dried using a lyophilizer at −80 °C and the stock solution of each extract was investigated for further biological activities. After extraction, IGB (10 g) was dissolved in ethanol and subjected to silica gel (60–120 mesh) column chromatography, using a gradient of CHCl_3_-EtOH (from 100:0 to 1:3), and was separated into eight fractions (Fr.1–Fr.8). Fr.1 was purified by preparative TLC (EtOH: chloroform, 1: 10) to obtain compound **1** (130 mg; yield: 1.38 %). Fr.3 was purified by preparative TLC (EtOH: chloroform, 1: 8) to obtain compound **2** (82 mg; yield: 0.82%) and compound **3** (45 mg; yield: 0.45%). Again, IGL (5 g) was dissolved in ethanol and subjected to silica gel (60–120 mesh) for column chromatography, using a gradient of n-Hexane-EtOAc (from 100:0 to 1:2), and was separated into nine fractions (Fr.1–Fr.9). The Fr.2 was purified by preparative TLC using Hexane-EtOAc, 10:1 to obtain compound **4** (80 mg; yield: 1.60%). The Fr.4 was purified by preparative TLC using Hexane-EtOAc, 7:1 to obtain compound **5** (70 mg; yield: 1.40%). Compound **1** was also obtained from IGW by column chromatography using the same protocol as in IGB.

### 2.4. Spectral data of isolated compounds

Chemical structure of purified compound was analyzed by ^1^H, ^13^C-NMR, FT-IR and HRMS. Compound (**1–5)** showed the following characteristics:

#### 6-allyl-6-(3-methylbut-2-en-1-yl)benzo[*d*][1,3]dioxol-5(6*H*)-one (1)

Colorless oil (130 mg; yield: 1.38%), ^1^H NMR (500 MHz, CDCl_3_): δ_H_ 5.74 (1H s, H-15), 5.73 (1H, s, H-15), 5.52 (1H, s, H-6), 5.49 (1H, m, H-8), 5.35 (1H, s, H-3), 4.93 (dd, *J* = 1.5, 17Hz, H-9), 4.87 (dd, *J* = 1.5, 10.5Hz, H-9), 4.85 (1H, m, H-11), 2.53 (1H, dd, *J* = 13.5, 7 Hz, H-7), 2.44 (1H, dd, *J* = 14.0, 7Hz, H-10), 2.17 (1H, dd, *J* = 14.0, 7Hz, H-10), 2.13 (1H, dd, *J* = 13.5, 7.5Hz, H-7), 1.55 (3H, s, H-13), 1.50 (3H, s, H-14); ^13^C NMR (125 MHz, CDCl_3_): δ_C_ 202.50 (C-1), 164.15 (C-5), 143.95 (C-4), 134.86 (C-12), 133.09 (C-8), 118.38 (C-11), 118.06 (C-9), 108.78 (C-3), 101.35 (C-15), 99.49 (C-6), 54.07 (C-2), 44.71 (C-7), 39.26 (C-10), 25.89 (C-13), 18.02 (C-14); FT-IR V_max_ (KBr, CHCl_3_, cm^−1^): 3078.6, 2975.3, 2917.5, 2857.5, 1726.1, 1505.4; HR-MS (+ESI) for C_15_H_18_O_3_ [M+H]^+^ at 247.1451 (calcd for C_15_H_19_O_3_: 247.1289). Elemental analysis: Found: C, 72.87%; H, 7.25%, O, 18.77%; C_15_H_18_O_3_ requires C, 73.15%; H, 7.37%, O, 19.49%.

#### Illicinone G (2)

Colorless oil (82 mg; yield: 0.82%), ^1^H NMR (500 MHz, CDCl_3_): δ_H_ 6.68 (1H, s, H-3), 5.76 (1H, m, H-8), 5.59 (1H, s, H-6), 5.52 (2H, d, *J* = 4.5 Hz, H-15), 5.09 (1H, d, *J* = 1.6 Hz, H-10), 5.05 (1H, d, *J* = 1.7 Hz, H-11), 4.92 (dd, *J* = 17, 2 Hz, H-9), 4.85 (dd, *J* = 10, 2 Hz, H-9), 3.03 (2H, d, *J* = 6.5 Hz, H-7), 1.18 (3H, s, H-13), 1.18 (3H, s, H-14); ^13^C NMR (125 MHz; CDCl_3_) δ_C_ 186.57 (C-1), 173.78 (C-5), 140.76 (C-3), 139.28 (C-11), 134.42 (C-8), 133.92 (C-2), 121.72 (C-10), 117.90 (C-9), 98.43 (C-6), 98.12 (C-15), 79.82 (C-4), 71.79 (C-12), 33.39 (C-7), 24.48 (C-13), 22.5 (C-14); FT-IR V_max_ (KBr, CHCl_3_, cm^−1^): 3440.0, 3079.4, 2961.3, 2924.5, 2851.8, 1727.8, 1678.0, 1651.9, 1625.8, 1463.0, 1260.6, 1187.9, 1096.6, 1029.7, 911.3, 801.0, 666.0; HR-MS (+ESI) for C_15_H_18_O_4_ [M+H]^+^ at m/z 263.1531 (Calcd for C_15_H_19_O_4_ 263.1239). Elemental analysis: Found: C, 68.72%; H, 6.77%, O, 24.33%; C_15_H_18_O_4_ requires C, 68.69%; H, 6.92%, O, 24.40%.

#### 1-Eicosene (3)

Colorless oil (45 mg; yield:0.45%), ^1^H-NMR (500 MHz; CDCl_3_) δ_H_ 5.74 (1H, m, H-2), 4.92 (1H, dd, *J* = 1.5, 17.0 Hz, H-1), 4.86 (1H, dd, *J* = 1.5, 10.5 Hz, H-1), 1.96 (2H, m, H-3), 1.34 (s, 2H), 1.28 (s, 2H), 1.22 (overlapping, 4H), 1.18 (overlapping, 24H), 0.88 (3H, t, *J* = 7Hz, H-20); ^13^C-NMR (125 MHz; CDCl_3_) δ_C_ 139.30 (C-2), 114.08 (C-1), 33.84 (C-3), 31.94 (C-18), 29.71 (C-5), 29.68 (C-6), 29.64 (C-4), 29.53 (C-7), 29.38 (C-8 to C-16), 29.17 (C-17), 22.71 (C-19), 14.14 (C-20); FT-IR V_max_ (KBr, CHCl_3_, cm^−1^): 3078.0, 2960.8, 2914.0, 2852.8, 1731.5, 1651.9, 1642.1, 1508.9, 1463.7, 1260.5, 1216.7; HR-MS (+ESI) for C_20_H_40_ [M+H]^+^ at m/z 281.2034 (M+H)^+^ (Calcd for C_20_H_41_ 281.3164). Elemental analysis: Found: C, 85.61%; H, 14.39%; C_13_H_26_ requires C, 85.63%; H, 14.37%.

#### 1-allyl-3,5-dimethoxy-4-(3-methylbut-2-enyloxy)benzene (4)

Colorless oil (80 mg; yield: 1.60%), ^1^H-NMR (500 MHz; CDCl_3_) δ_H_ 6.40 (2H, s, H-2, H-6), 5.97 (1H, m, H-8), 5.58 (1H, tsep, *J* = 7, 3, 1.5 Hz, H-11), 5.09 (2H, m, H-9), 4.46 (2H, d, *J* = 5 Hz, H-10), 3.84 (6H, s, 2-OCH_3_), 3.34 (2H, d, *J* = 5 Hz, H-7), 1.75 (3H, d, *J* = 1 Hz, H-13), 1.67 (3H, d, *J* = 1 Hz, H-14); ^13^C-NMR (125 MHz; CDCl_3_) δ_C_ 153.52 (C-3, C-5), 138.24 (C-12), 137.32 (C-8), 135.64 (C-1), 120.87 (C-11), 115.95 (C-9), 105.33 (C-2, C-6), 69.52 (C-10), 56.02 (2-OCH_3_), 40.57 (C-7), 29.71 (C-13), 25.85 (C-14); FT-IR V_max_ (KBr, CHCl_3_, cm^−1^): 2924.9, 2853.8, 1737.0, 1674.5, 1639.0, 1589.6, 1505.5, 1456.6, 1239.6, 1130.2, 977.9, 913.0, 821.6, 665.9; HR-MS (+ESI) for C_16_H_22_O_3_ [M+H]^+^ at m/z 263.1465 (Calcd for C_16_H_23_O_3_ 263.1602). Elemental analysis: Found: C, 73.17%; H, 7.88%, O, 18.23%; C_16_H_22_O_3_ requires C, 73.25%; H, 8.45%, O, 18.30%.

#### Tridec-1-ene (5)

Colorless oil (70 mg; yield 1.40%), ^1^H-NMR (500 MHz; CDCl_3_) δ_H_ 5.75 (1H, m, H-2), 4.92 (1H, dd, *J* = 2, 17.0 Hz, H-1), 4.86 (1H, dd, *J* = 2, 10Hz, H-1), 1.96 (2H, m, H-3), 1.27–1.26 (18H, m, overlapping signals), 0.88 (3H, t, *J* = 6.5 Hz, H-13); ^13^C-NMR (125 MHz; CDCl_3_) δ_C_ 138.96 (C-2), 113.73 (C-1), 33.49 (C-3), 31.58 (C-11), 29.36 (C-5), 29.32 (C-6), 29.28 (C-4), 29.17 (C-7), 29.02(C-8), 28.82(C-9), 28.60 (C-10), 22.35 (C-12), 13.79 (C-13); FT-IR V_max_ (KBr, CHCl_3_, cm^−1^): 3079.7, 2960.5, 2914.0, 2855.0, 1777.2, 1630.2, 1630.2, 1493.9; HR-MS (+ESI) for C_13_H_26_ [M+H]^+^ at m/z 183.0038 (Calcd for C_13_H_27_ 183.2068). Elemental analysis: Found: C, 85.45%; H, 14.55%; C_13_H_26_ requires C, 85.63%; H, 14.37%.

### 2.5. Measuring cell viability

The cell lines A549 and MIAPaCa2 were cultured in respective complete media DMEM, Ham’s F12k and MEM, respectively and supplemented with 10% Foetus Bovine Serum, 1% Gentamycin (antibiotics), 10% Penstrep. However, cells (1 **×** 10^6^ per mL) were seeded in tissue culture grade multi-well plates in complete medium. The plates were incubated under standard conditions in 37 **°C humidified atmosphere containing 5% CO**_2_ for 24 h. After incubation, the whole medium was replaced with FBS free medium and incubated overnight. Afterwards, the cells were treated with the samples of *I. griffithii* in different concentrations in each well and incubated for 24 h. Well holding medium alone (untreated cells) served as a control.

By using in vitro MTT assay, the cytotoxicity study was evaluated. At first, each well was mixed with 10 mL of MTT (5 mg/mL), and incubated for 4 h. After observation of dark purple crystals of formazan at the bottom of the wells by an inverted microscope, 0.04 N HCl with Isopropanol (100 mL) was mixed to each well and prepared the solution suitable for absorbance measurement. The effect of the samples on the proliferation of cells was expressed as the % cell viability. The half maximal inhibitory concentration (IC_50_) of the samples was determined from the dose response plotted curves [21].

### 2.6. Antimicrobial assay

#### 2.6.1. General

The extracts and isolated compounds were tested for antimicrobial activity against microorganisms using the reported method [22]. Four microbes *Escherichia coli* (ATCC^®^11229^™^), *Staphylococcus aureus* (ATCC^®^11632^™^), *Pseudomonas aeruginosa* (ATCC^®^27853^™^), and *Candida albicans* (ATCC^®^90028^™^) for antimicrobial activity were purchased from HiMedia. Mueller Hinton Agar (MHA) media was prepared by dissolving 3.8 g of MHA in 100 mL distilled water. After solidification of the media, inoculums of approximately 1 × 10^8^ CFU/mL were spread over the plate. Then, 6mm wells were made in the solidified media using a cork borer. Hundred μL of test samples were poured into the required wells and plates were incubated. For bacteria, incubation time is 48 h at 37 °C and for fungus 18 h at 30 °C. The experiment was carried out in triplicates. Antimicrobial activities were evaluated by measuring the zone of inhibition against test microbes and results were presented as mean.

#### 2.6.2. Minimum inhibitory concentration (MIC)

Minimum inhibitory concentration was determined by resazurin reduction assay in 96 well microtitre plates (Nunc™, Thermo Fisher Scientific Inc). At first 100 μL of sterile broth was added to all 96 wells and test samples were serially diluted. Then 20 μL of bacterial and fungal suspension was added to each well to achieve a concentration of 5 × 10^5^CFU/mL. Finally, 20 μL of resazurin indicator was added to each well. Plates were incubated at 30 °C for 18–42 h and colour change was then assessed visually. The plates were observed visually for the colour change of the indicator. The colour change from purple to colourless indicates the growth of microbes. MIC of test samples was determined as the lowest concentration of the compound at which no microbial growth was observed [23].

## 3. Results and discussions

**Figure 1:**
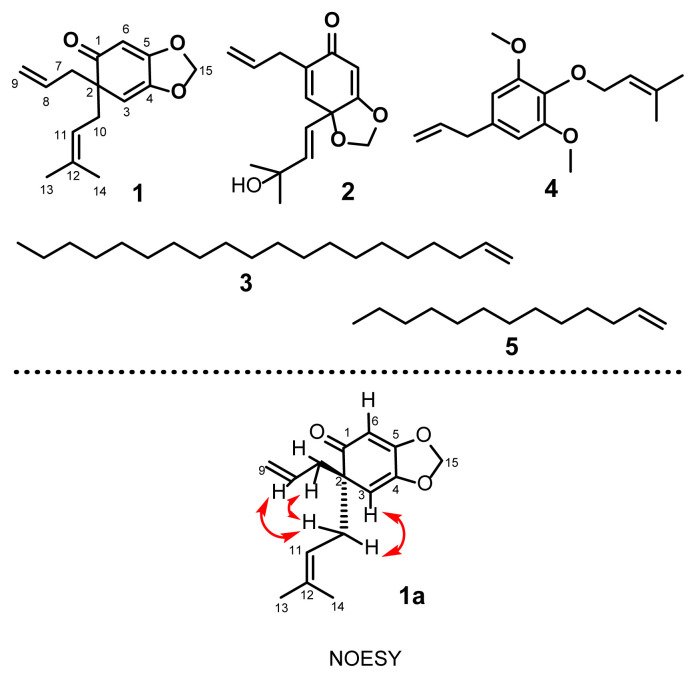
Structure of isolated compounds **(1–5).**

In this study, we describe the isolation of five compounds (**1**–**5**) from *I. griffithii* as well as cytotoxicity and antimicrobial activity (Figure 1). Compound **1** was obtained as colourless oil from ethanol extracts of bark. Its molecular formula was determined as C_15_H_18_O_3_ by elemental analysis and HR-MS with m/z 247.1451 for pseudo-molecular ion [M+H]^+^. In FT-IR spectrum, the absorption band observed at 1726.1 cm^−1^ is due to the presence of a carbonyl group in the structure. In the ^1^H NMR spectrum, the two singlets at δ 5.74 and 5.73 ppm each integrated into one proton are assigned to a methylenedioxy group. Further, two singlets that appeared at δ 5.52 and 5.35 ppm are assigned to H-6 and H-3 protons of C_6_ ring respectively. Two-three proton singlets at δ 1.50 and 1.55 were assigned to methyl connected to a double bond. The double doublet signals at δ 2.17 with *J* = 14.0 and 7 Hz and 2.44 ppm with *J* = 14.0 and 7 Hz each integrating to one proton attributed to a methylene group attached to a prenyl moiety. The presence of an allyl moiety was indicated by two single proton signals at δ 2.13 with *J* = 13.5 and 7.5 Hz and 2.53 ppm with *J* = 13.5 and 7 Hz and two olefinic signals at δ 4.87 with *J* = 1.5 and 10.5 Hz and 4.93 ppm with *J* = 1.5 and 17 Hz along with a multiplate at 5.49 ppm integrated to one proton. Thus, this proton spectrum is very similar to the compound isolated from *I. anisatum* as reported in the literature [25]. The structure of compound **1** is further confirmed by its ^13^C NMR spectrum. The presence of a carbonyl group was confirmed by ^13^C NMR signal at δ 202.50 ppm and olefinic carbons are confirmed by δ 18.02, 25.89, 39.26, 44.71, 133.09, 118.06, 118.38, 134.86 ppm. Complete assignment of proton and carbon signals of compound **1** was not done so far. Therefore, we have thoroughly studied the compound using 2D NMR spectra. The complete proton-proton coupling network was determined by its ^1^H-^1^H COSY NMR experiment. The proton-carbon correlation of the molecule was also established with HSQC experiment. The NOESY spectrum showed the correlations between H-10 methylene (δ_H_ 2.17) of the prenyl group with the H-3 (δ_H_ 5.35) of the C_6_ ring. In addition, H-10 also correlated with the H-7 methylene (δ_H_ 2.13) and H-8 methine (δ_H_ 5.49) of the allyl group. The sign of specific rotation of **1** ([α]_D_^31.8^ = −0.169) was found negative which was opposite to the reported compound ([α]_D_^24^ = + 5.64) [24]. Its absolute configuration was assigned as 2S. On the basis of this evidence, the structure of the molecule was confirmed as 6-allyl-6-(3-methylbut-2-en-1-ylbenzo[*d*][1,3]dioxol-5(6*H*)-one (**1**).

Compound **2** was obtained as colourless oil. Its molecular formula, C_15_H_18_O_4_, as determined by elemental analysis and HR-MS with m/z 263.1531 for pseudo-molecular ion [M+H]^+^. IR absorptions revealed the presence of carbonyl group at 1727.8 cm^−1^ and a hydroxyl group at 3440.0 cm^−1^. In the ^1^H NMR spectrum, two singlets appear at δ_H_ 6.68 and 5.59, each integrated into one proton, due to two aromatic H-3 and H-6 protons respectively. A doublet signal displayed at δ_H_ 5.52 is due to the presence of a methylenedioxy group. Allyl moiety showed signals including a multiplet at δ_H_ 5.76 integrating a proton at H-8 position, two doublets of doublets at δ_H_ 4.85 and 4.92 integrating two H-9 protons, a doublet at δ_H_ 3.03 integrating two H-7 protons. The ^13^C NMR signals at δ_C_ 33.39, 133.92, and 117.90 confirm the presence of allyl moiety. The presence of a carbonyl group was confirmed by ^13^C NMR signal at δ_C_ 186.57. Based on this evidence and by comparison with the spectral data of the compound reported from *I. tashiroi* [26], the structure of the molecule was confirmed as Illicinone G (**2**).

Compound **3** was obtained as colourless liquid with molecular formula C_20_H_40_ as determined by elemental analysis and HRESIMS with m/z 279.0836 for pseudo molecular ion [M-H]^+^. The ^1^H NMR, ^13^C NMR data exhibited the presence of a double bond at C-1 due to one methyne [δ_H_ 5.74 (m, 1H); δ_C_ 139.20], a methylene [δ_H_ 4.92 (1H, dd, *J* = 1.5, 17.0Hz, H-1a), 4.86 (1H, dd, *J* = 2.5, 10.5Hz, H-1b), δ_C_ 113.96] and one methyl at [δ_H_ 0.88 (t, 3H, *J* = 7Hz); δ_C_ 14.03], suggesting that compound **5** is long-chain alkene. The structure was confirmed by comparison with the spectral data of literature determined as 1-Eicosene (**3**) [27].

Compound **4** was obtained as colourless oil. Its molecular formula, C_16_H_22_O_3_, as determined by elemental analysis and HR-MS with m/z 263.1465 for pseudo-molecular ion [M+H]^+^. The IR spectra displayed an absorption band attributable to the methoxy group at 2853.8 cm^−1^. The NMR spectrum exhibited the presence of two methoxy groups at δ_H_ 3.84 and δ_C_ 56.02. The ^1^H NMR data indicated the presence of two aromatic protons H-2 and H-6 at δ_H_ 6.40. An allyl group showed signals including a multiplet at δ_H_ 5.97 integrating to one proton of H-8, a doublet at δ_H_ 5.09 integrating to two protons at H-9, a doublet at δ_H_ 3.34 integrating to two protons at H-7. The presence of a prenyl moiety displayed signals including two singlets at δ_H_ 1.67 and 1.75 for two methyls, a doublet at δ_H_ 4.46 integrating to two protons at H-10 and a triplet of septets at δ_H_ 5.58 integrating to one proton at H-11. The structure was confirmed by comparison with the spectral data of the compound reported from *I anisatum* [25] as 1-allyl-3,5-dimethoxy-4-(3-methylbut-2-enyloxy)benzene (**4**).

Compound **5** was obtained as colourless liquid with a molecular formula C_13_H_26_ as determined by elemental analysis and HR-MS with m/z 183.0038 for pseudo-molecular ion [M+H]^+^. The ^1^H NMR, ^13^C NMR data exhibited the presence of a double bond at C-1 due to one methyne [δ_H_ 5.75 (m, 1H); δ_C_ 138.86], a methylene [δ_H_ 4.92 (1H, dd, *J* = 2, 17.0 Hz,


$$\%\;\text{cell viability}=\frac{\text{Absorbance of treated cells}}{\text{Absorbance of control cells}}\times 100$$

H-1), 4.86 (1H, dd, *J* = 2, 10Hz, H-b), δ_C_ 113.63] and one methyl at [δ_H_ 0.88 (t, 3H, *J* = 6.5 Hz); δ_C_ 13.70], suggesting that compound **5** is long-chain alkene. The structure was confirmed by comparison with the spectral data of literature determined as Tridec-1-ene (**5**) [28]. In this study, two prenylated C_6_-C_3_ (**1, 2**) and one olefin (**3)** were isolated from the bark, and one phenolic (**4**) & one long chain hydrocarbon **5** were isolated from the leaf of *I griffithii.* Prenylated compound **1** was also isolated from wood of *I griffithii*. All five compounds were isolated from this *Illicium* species for the first time.

**Figure 2:**
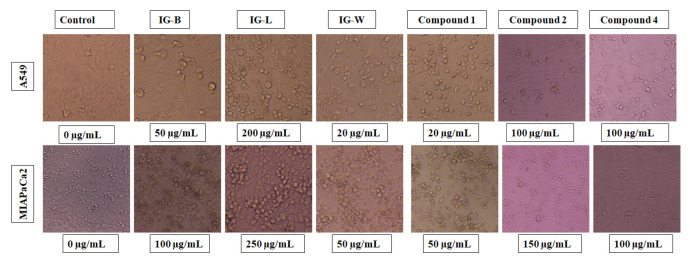
Photomicrograph of cells showing morphological changes/death induced by 24 h treatment with *I. griffithii* in comparison to control.

The cytotoxicity of *I. griffithii* was established by MTT assay against lung cancer (A549) and pancreatic cancer (MIAPaCa2) cell lines. The control cells showed high proliferation that has been taken as 100%. There is a decrease in the percentage of viable cells with an increase in doses of samples. A treatment with a 100 μg/mL dose of Compound **1** to A549 and MIAPaCa2 cells shows a significant decrease in the viable cells by almost −5.176% and 2.021% respectively at 24 h. Compound **1** induced significant cytotoxicity against A549 and MIAPaCa2 with IC_50_ values of 15.01 ± 2.69 μg/mL and 47.77 ± 2.38 μg/mL, respectively. The similar types of compounds (**2, 4**) exhibited moderate activity against A549 and MIAPaCa2 with IC_50_ values ranging from 67.69–240.42 μg/mL and 74.60–262 μg/mL, respectively. For long-chain hydrocarbons compound **3** & **5** exhibited moderate anticancer activity against A549 and MIAPaCa2 with IC_50_ 240.42 ± 0.54 μg/mL; 262.00 ± 2.03 μg/mL and 174.12 ± 1.12; 292.25 ± 0.92 μg/mL, respectively. Earlier, we have found that such types of compounds inhibit A549 lung cancer cells through PI3K pathway inhibition. So, it may be possible that these compounds from this plant showed anticancer activity through AA pathways of PI3 kinase pathway of cancer growth [23]. The results were compared with standard Doxorubicin with IC_50_ values of 1.62 ± 1.86 and 0.47 ± 2.63 against A549 and MIAPaCa2 respectively (Table 1). The cell lines have shown morphological changes in 24 h treatment induced by samples in comparison to control (Figure 2).

**Figure 3:**
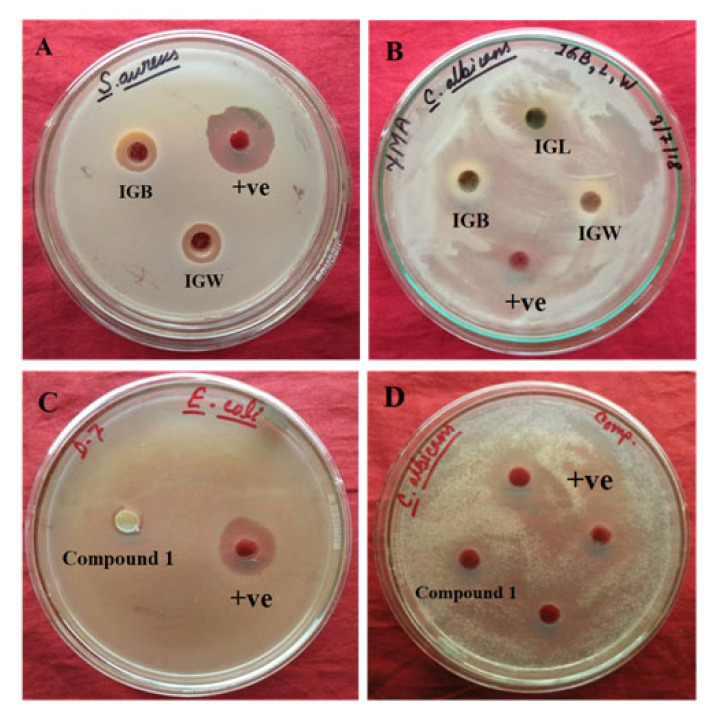
Antimicrobial activity of *I. griffithii* extracts against (A) *S. aureus* with Rifampicin +ve control, (B) *C. albicans* with Fluconazole +ve control; Compound **1** against (C) *E. coli* with Amikacin +ve control, (D) *C. albicans* with Fluconazole +ve control.

The study of antimicrobial activity of the *I. griffithii* against four microbes *E. coli*, *C. albicans*, *S. aureus*, and *P. aeruginosa* by Agar well diffusion method (Table 2). Among these microbes, *E. coli* and *C. Albicans* were found to be sensitive for the compounds (**1–5**), whereas the extracts were exhibited activity against *S. aureus* and *P. aeruginosa*. The compound **1** is appeared to be most active with zone of inhibition (ZOI) 9.16 ± 0.28 mm and 12.50 ± 0.50 mm against *E. coli* and *C. albicans* respectively compared with standards Amikacin and Fluconazole (Table 3). The MIC values of compound **1** are 7.50 ± 0.28 and 7.50 ± 0.86 μg/mL against *E. coli* and *C. albicans* whereas MIC values of Amikacin and Fluconazole are 4.37 ± 0.50 and 7.50 ± 0.36 μg/mL, respectively (Figure 3). Similarly, compounds **2** and **4** displayed moderate activity against *E. coli* with ZOI = 6.23 ± 0.50 mm and 8.36 ± 0.22 mm and against *C. albicans* with ZOI = 8.50 ± 0.46 mm and 7.45 ± 0.34 mm, respectively. In our earlier study, we have found that long-chain hydrocarbons demonstrated antimicrobial activities [19,23]. Here, two long-chain alkene compounds **3** and **5** showed moderate activity against *C. albicans* with ZOI = 9.15 ± 0.26 mm and 8.45 ± 0.37 mm, respectively. To the best of our knowledge, this is the first study of isolation of compounds from bark, wood, and leaf of *I. griffithii* along with cytotoxicity and antimicrobial activities from North East India.

## 4. Conclusion

Herein, we have studied isolated five bioactive compounds from the leaves and bark of *I. griffithii*, an endemic plant of Eastern Himalayan Biodiversity Mega Center in North East India. Compound **1** exhibited significant cytotoxicity against A549 lung cancer and MIAPaCa2 pancreatic cell lines with IC_50_ 15.01 ± 2.69 μg/mL and 47.77 ± 2.38 μg/mL, respectively. Further, compound **1** exhibited excellent antimicrobial activity against *E. coli* and *C. albicans* with MIC 7.50 ± 0.28 μg/mL and 7.50 ± 0.86 μg/mL respectively as compared to the standard marketed drug Amikacin (4.37 ± 0.50 μg/mL) and Fluconazole (7.50 ± 0.36 μg/mL). Moreover, the other isolated compounds also exhibited promising potential for applications as anticancer and antimicrobial agents. Thus, this is the first report of cytotoxicity and antimicrobial activities of the isolated compounds from bark, wood, and leaf of *I. griffithii*, an endemic plant of North East India. Hence, our study suggests that the *I. Griffithii* has great potential to be used as an effective natural therapy against cancer and microbial infection in near future.

## Supporting Information

Figure S1. Flow chart of isolation of compounds from *I. griffithii*.

Figure S2. ^1^H NMR (CDCl_3_, 500MHz) Spectra of Compound **1**.

Figure S3. ^13^C NMR (CDCl_3_, 125MHz) Spectra of Compound **1**.

Figure S4. HR-ESI-MS of Compound **1**.

Figure S5. ^1^H-^1^H COSY NMR (CDCl_3_, 500MHz) Spectra of Compound **1**.

Figure S6. ^1^H-^13^C HSQC NMR (CDCl_3_, 500MHz) Spectra of Compound **1**.

Figure S7. NOESY (CDCl_3_, 500MHz) Spectra of Compound **1**.

Figure S8. ^1^H NMR (CDCl_3_, 500MHz) Spectra of Compound **2**.

Figure S9. ^13^C NMR (CDCl_3_, 125MHz) Spectra of Compound **2**.

Figure S10. HR-ESI-MS of Compound **2**.

Figure S11. ^1^H NMR (CDCl_3_, 500MHz) Spectra of Compound **3**.

Figure S12. ^13^C NMR (CDCl_3_, 125MHz) Spectra of Compound **3**.

Figure S13. HR-ESI-MS of Compound **3**.

Figure S14. ^1^H NMR (CDCl_3_, 500MHz) Spectra of Compound **4**.

Figure S15. ^13^C NMR (CDCl_3_, 125MHz) Spectra of Compound **4**.

Figure S16. HR-ESI-MS of Compound **4**.

Figure S17. ^1^H NMR (CDCl_3_, 500MHz) Spectra of Compound **5**.

Figure S18. ^13^C NMR (CDCl_3_, 125MHz) Spectra of Compound **5**.

Figure S19. HR-ESI-MS of Compound **5**.

Figure S20. MIC of Compound 1 (E–F).

## Figures and Tables

**Table 1:** Cytotoxicity of the isolated compound and different extracts of *I. griffithii* against A549 and MIAPaCa2 cell lines^a^.

Sample	Cytotoxicity IC_50_ (μg/mL)	
	A549	MIAPaCa2
IG-B	34.70 ± 5.75	76.43 ± 1.41
IG-W	13.21 ± 2.61	42.84 ± 0.48
IG-L	185.68 ± 1.25	331.71 ± 0.86
Compound **1**	15.01 ± 2.69	47.77 ± 2.38
Compound **2**	75.11 ± 2.36	108.65 ± 0.93
Compound **3**	240.42 ± 0.54	262.00 ± 2.03
Compound **4**	67.69 ± 1.90	74.60 ± 2.12
Compound **5**	174.12 ± 1.12	292.25 ± 0.92
Doxorubicin	1.62 ± 1.86	0.47 ± 2.63

a IC_50_ is the half-maximal inhibitory concentration.

**Table 2:** Antimicrobial activity with Zone of Inhibition of extracts (mean ± SD) for different crude extracts and compounds of *I. Griffithii* againts Gram-positive and Gram-negative bacteria^a^.

Samples	Zone of Inhibition (ZOI) in mean ± SD (mm)			
	*E. coli* gram negative	*S. aureus* gram positive	*P. aeruginosa* gram negative	*C. albicans* Fungi
IGB	NF	12.66 ± 0.57	NF	NF
IGW	NF	10.10 ± 0.01	10.33 ± 0.45	NF
IGL	NF	NF	NF	10.60 ± 0.44
Compound **1**	9.16 ± 0.28	NF	NF	12.50 ± 0.51
Compound **2**	8.36 ± 0.22	NF	NF	7.45 ± 0.34
Compound **3**	NF	NF	NF	8.45 **±** 0.37
Compound **4**	6.23 ± 0.50	NF	NF	8.50 ± 0.46
Compound **5**	NF	NF	NF	9.15 **±** 0.26
Amikacin	19.33 ± 0.57	ND	ND	ND
Fluconazole	ND	ND	ND	15.90 ± 0.11
Rifampicin	ND	22.83 ± 1.04	ND	ND
Gentamicin	ND	ND	16.15 ± 0.17	ND

a ND: Not Determined, NF: Activity not found, ZOI = Zone of inhibition in mean **±** SD (mm).

**Table 3:** Minimum inhibitory concentration (MIC) of compound **1** isolated from *I. griffithii*^a^.

Reference microbes	MIC (μg/mL)		
	Compound 1	Amikacin	Fluconazole
*E. coli*	7.50 ± 0.28	4.37 ± 0.50	ND
*C. albicans*	7.50 ± 0.86	ND	7.50 ± 0.36

a ND: Not Determined, MIC = Minimum inhibitory concentration in μg/mL.
