# Supplementary figures and images for: Major chemical constituents from Illicium griffithii Hook. f. & Thoms of North East India and their cytotoxicity and antimicrobial activities
Source: Turk J Chem. 2022 Apr 8;46(5):1468–76. doi: 10.55730/1300-0527.3451 (PMC10390145; doi:10.55730/1300-0527.3451)

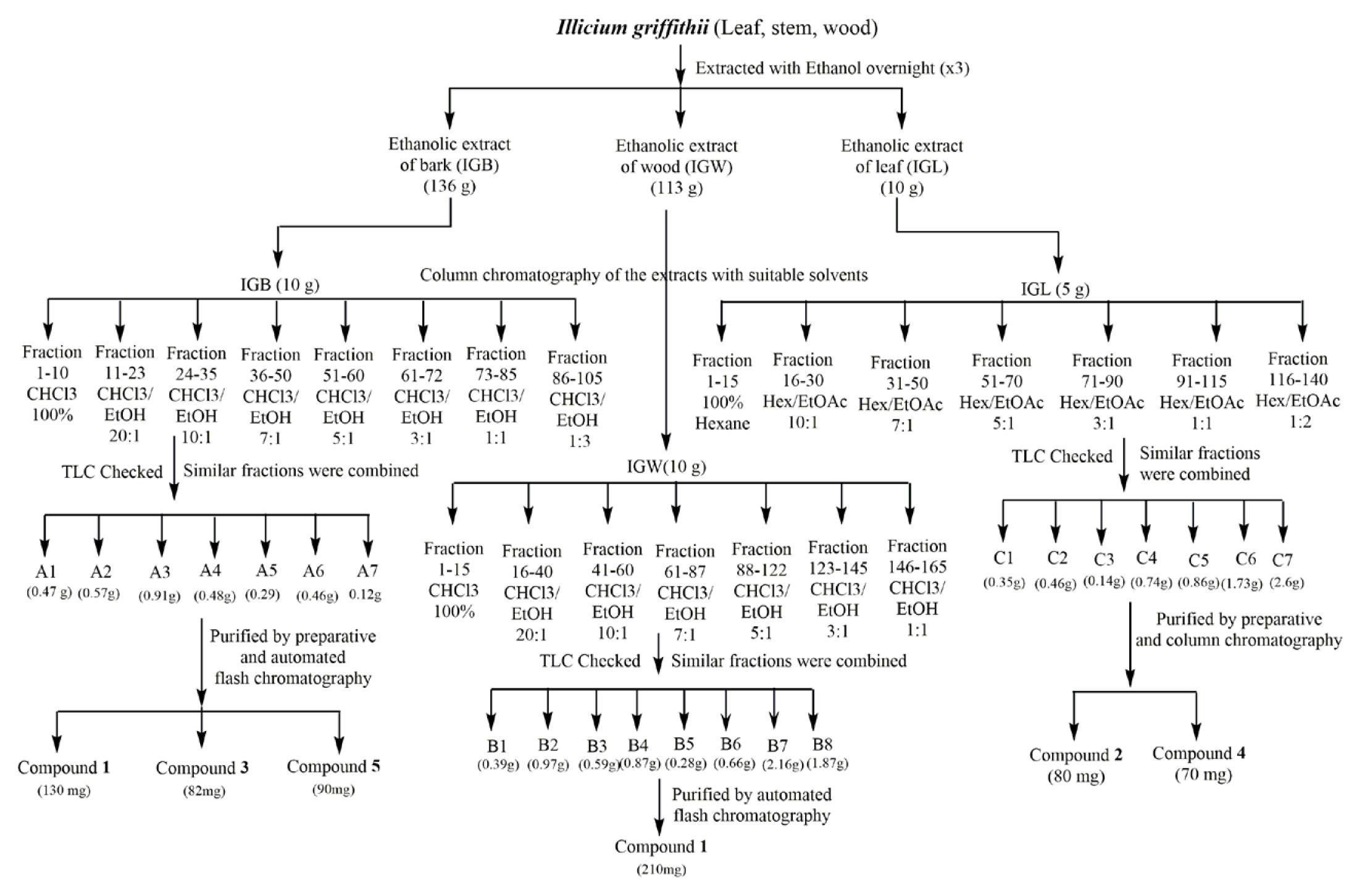

Supplement: Figure S1. — Flow chart of isolation of compounds from I. griffithii. [file turkjchem-46-5-1468s1.tif]

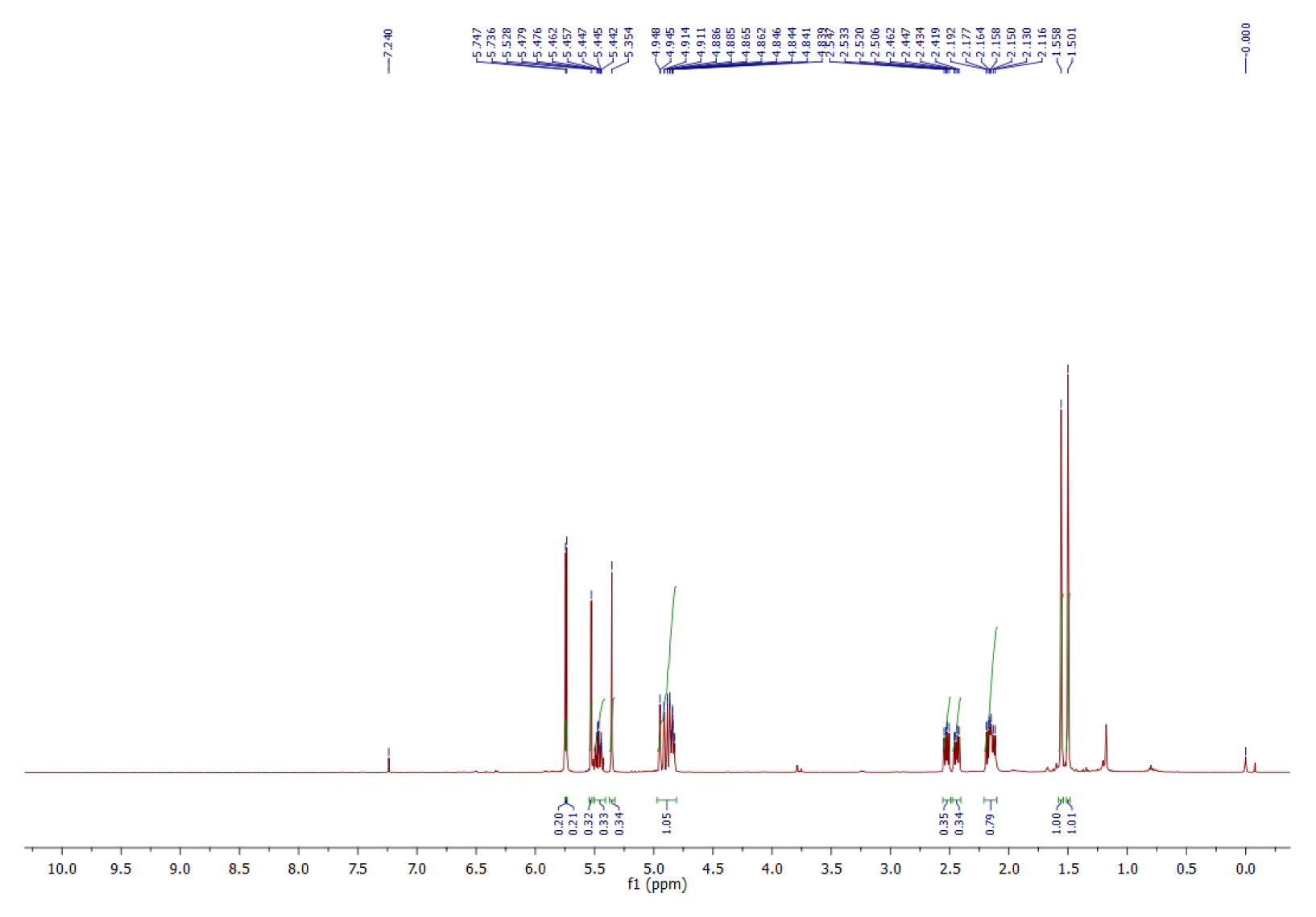

Supplement: Figure S2. — 1H NMR (CDCl3, 500MHz) Spectra of Compound 1. [file turkjchem-46-5-1468s2.tif]

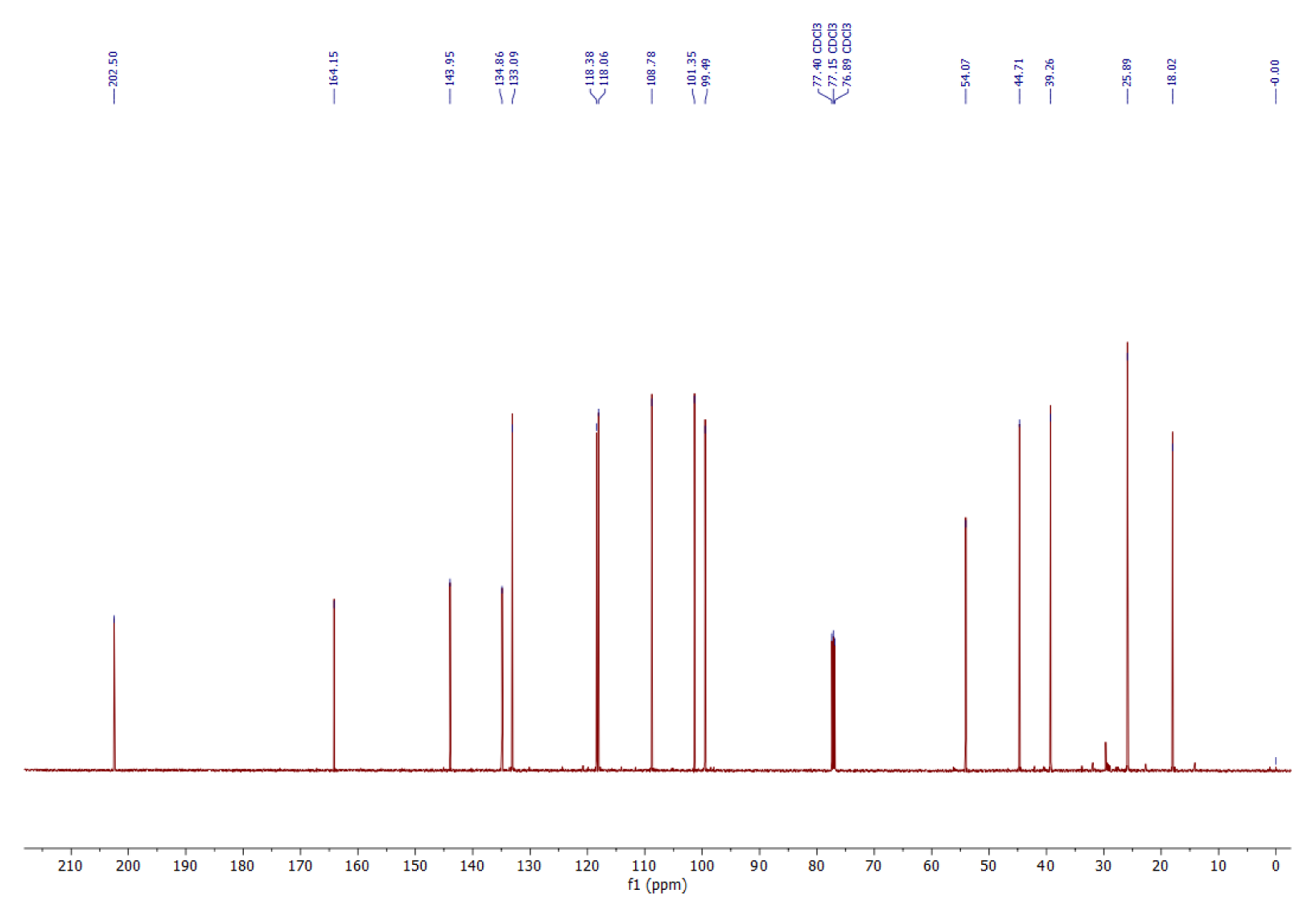

Supplement: Figure S3. — 13C NMR (CDCl3, 125MHz) Spectra of Compound 1. [file turkjchem-46-5-1468s3.tif]

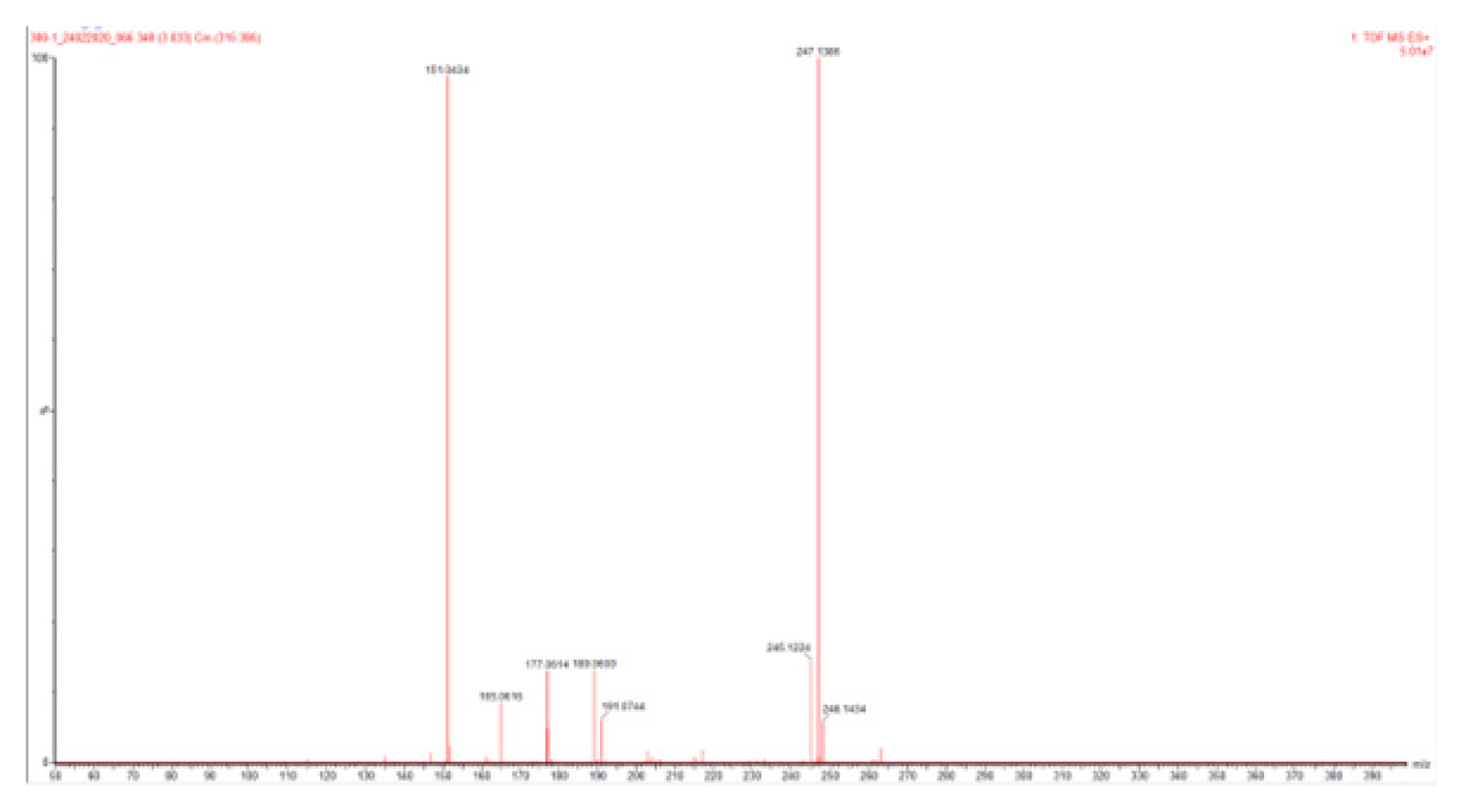

Supplement: Figure S4. — HR-ESI-MS of Compound 1. [file turkjchem-46-5-1468s4.tif]

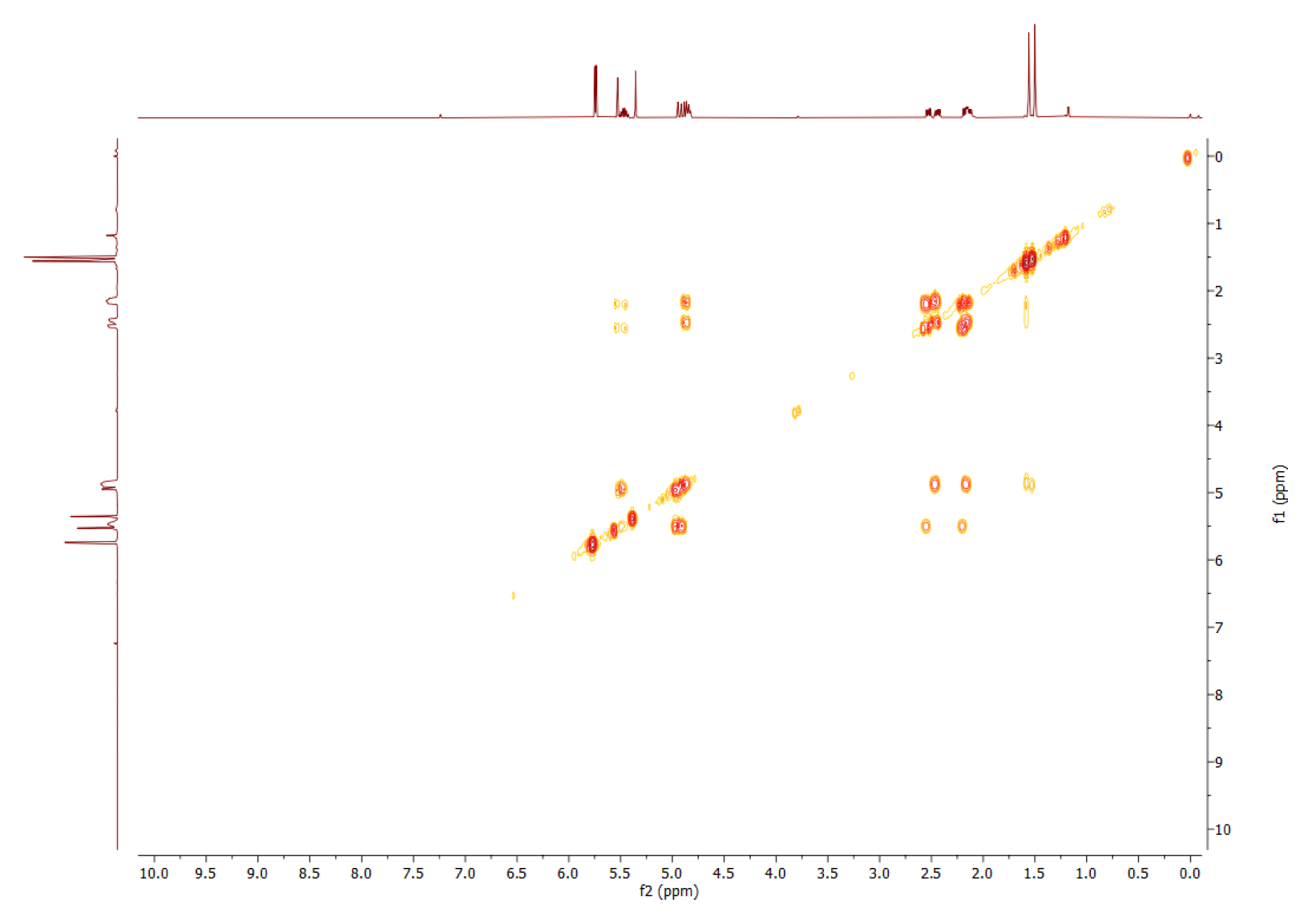

Supplement: Figure S5. — 1H-1H COSY NMR (CDCl3, 500MHz) Spectra of Compound 1. [file turkjchem-46-5-1468s5.tif]

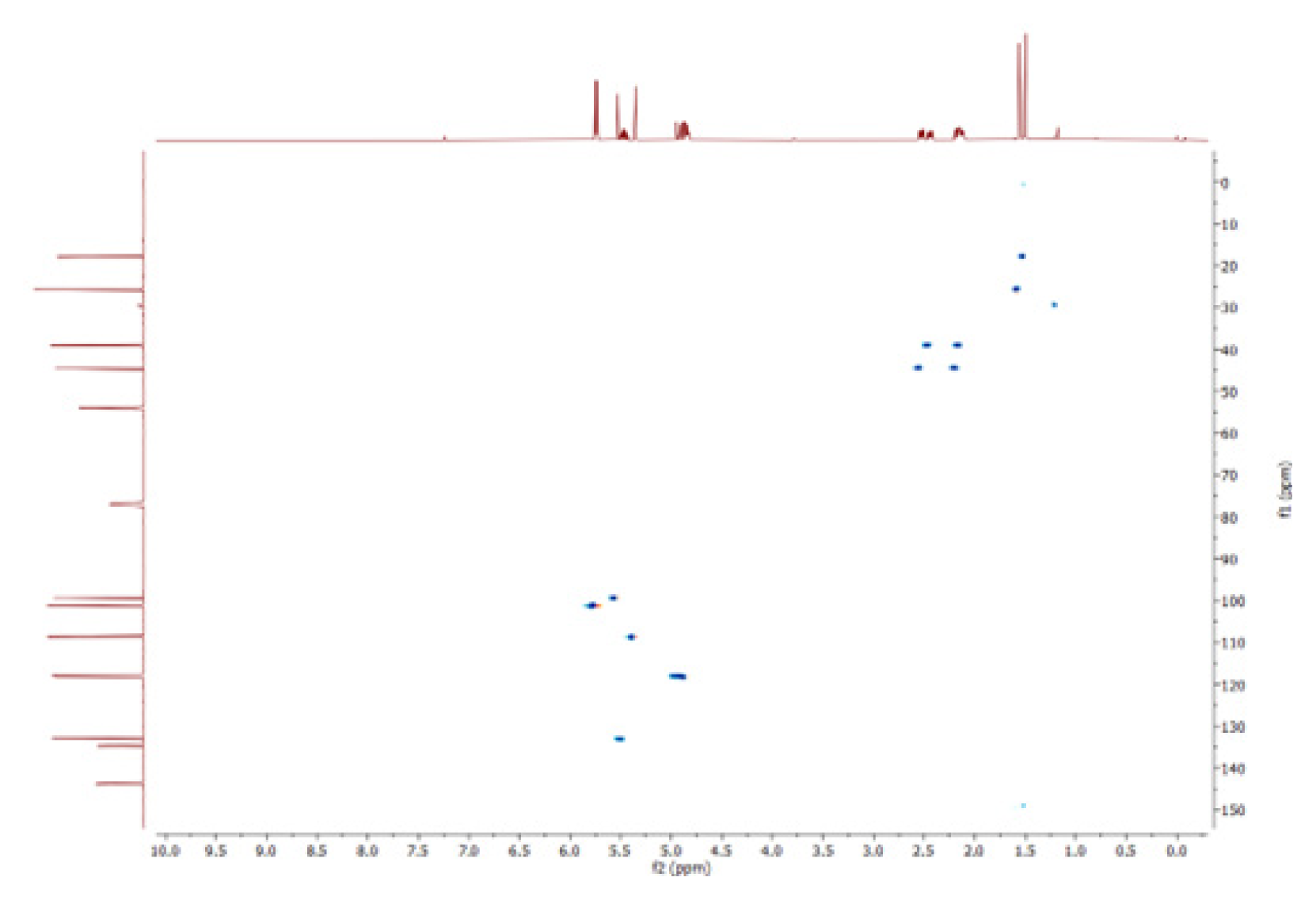

Supplement: Figure S6. — 1H-13C HSQC NMR (CDCl3, 500MHz) Spectra of Compound 1. [file turkjchem-46-5-1468s6.tif]

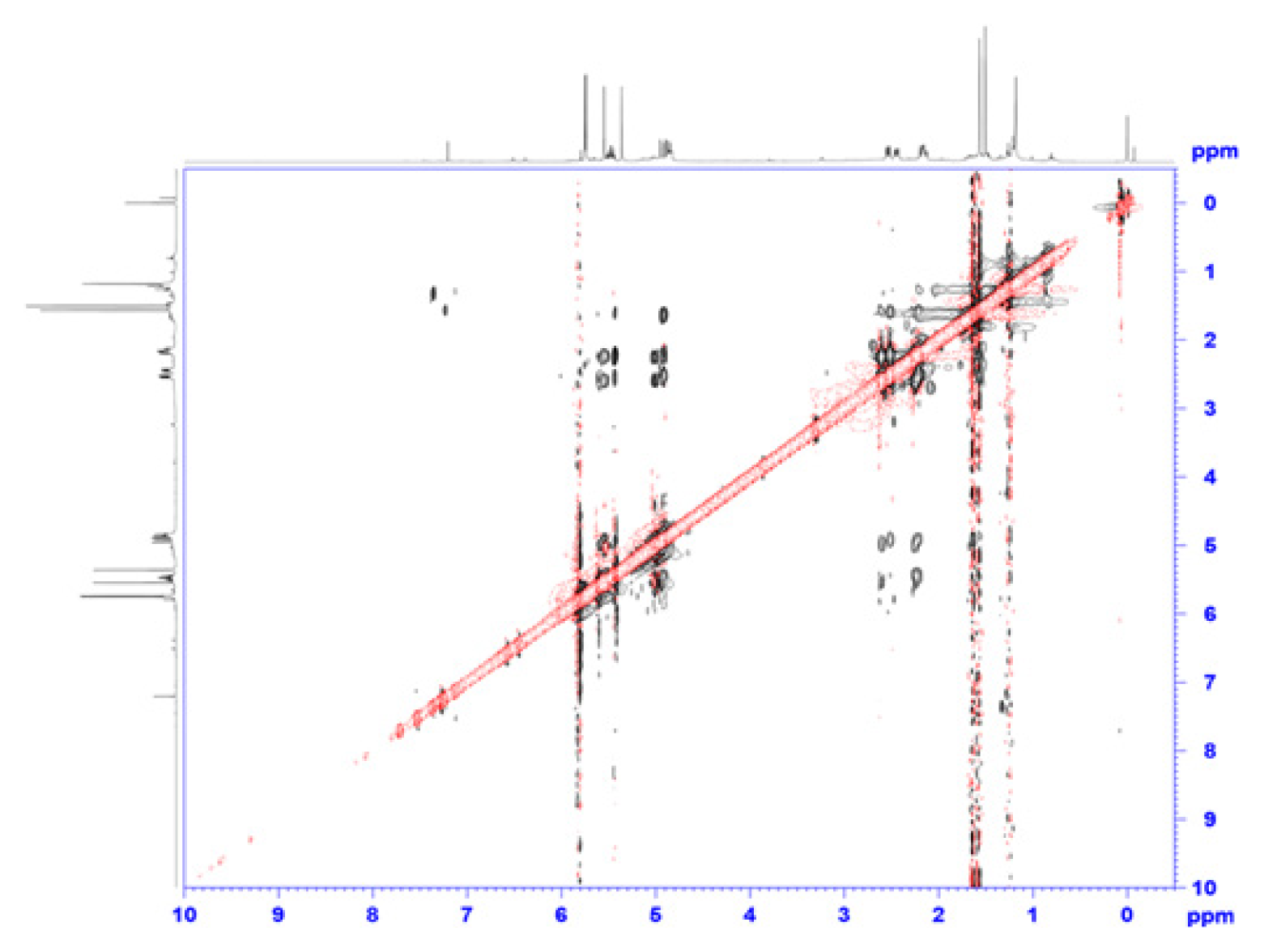

Supplement: Figure S7. — NOESY (CDCl3, 500MHz) Spectra of Compound 1. [file turkjchem-46-5-1468s7.tif]

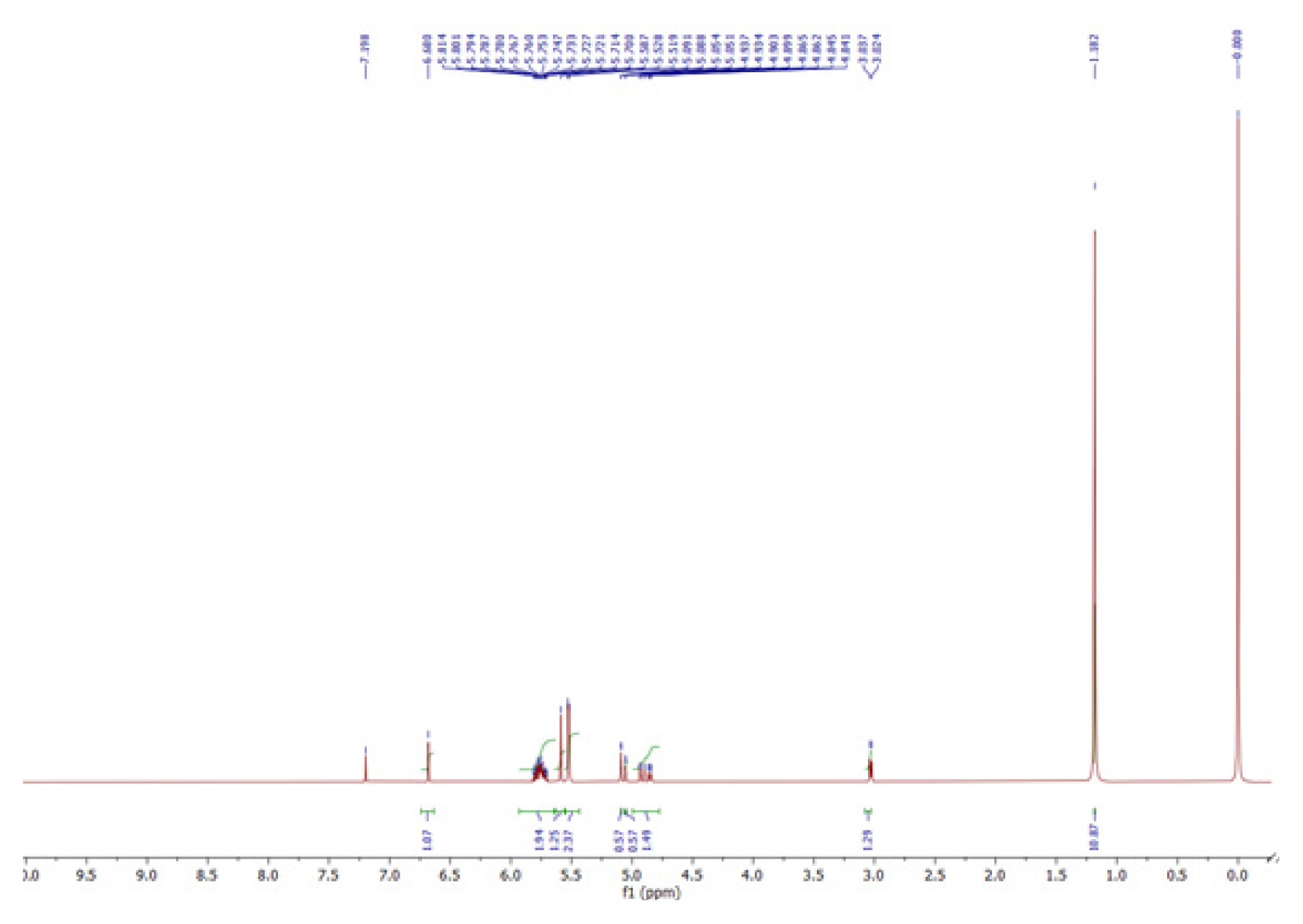

Supplement: Figure S8. — 1H NMR (CDCl3, 500MHz) Spectra of Compound 2. [file turkjchem-46-5-1468s8.tif]

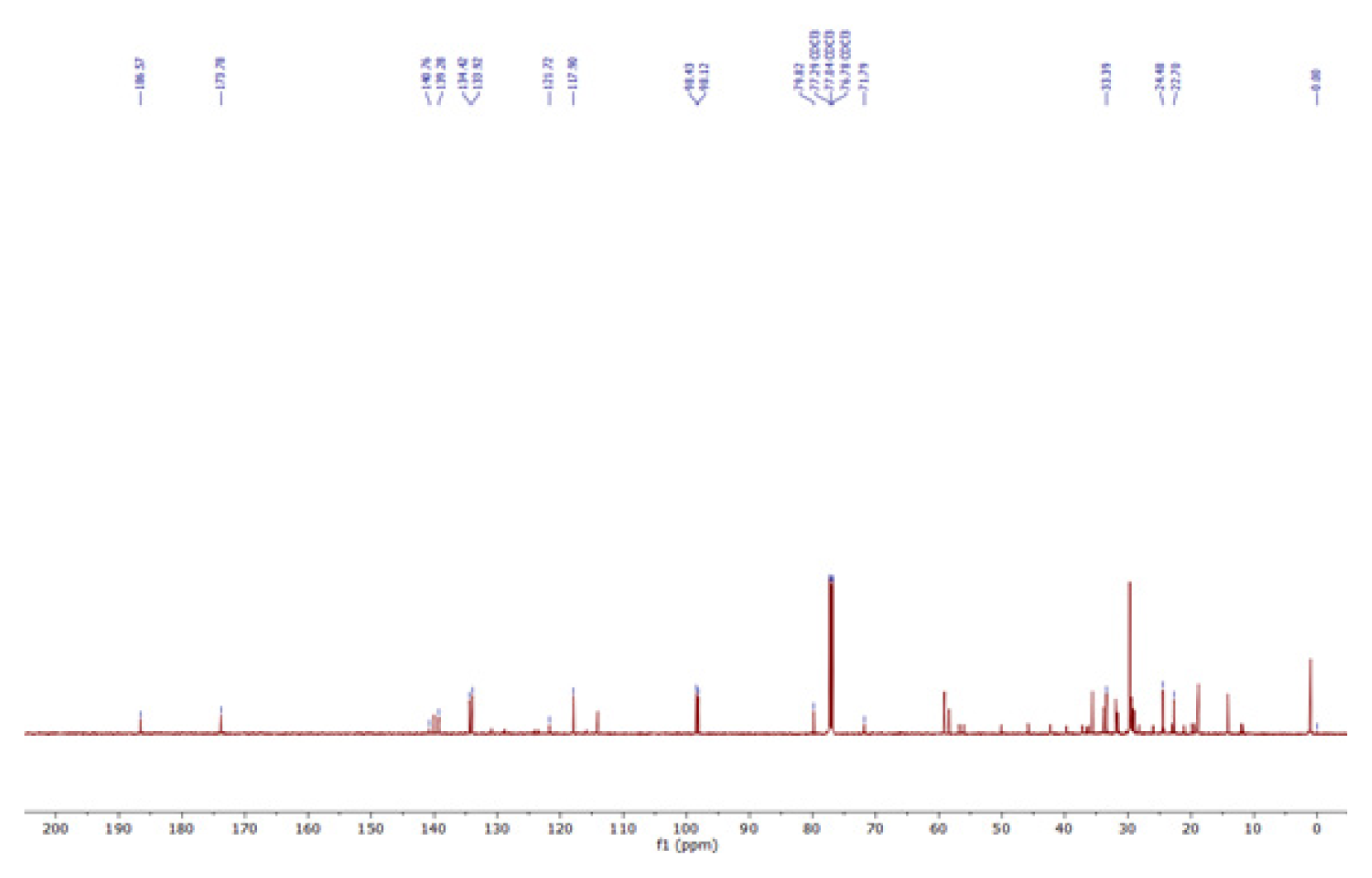

Supplement: Figure S9. — 13C NMR (CDCl3, 125MHz) Spectra of Compound 2. [file turkjchem-46-5-1468s9.tif]

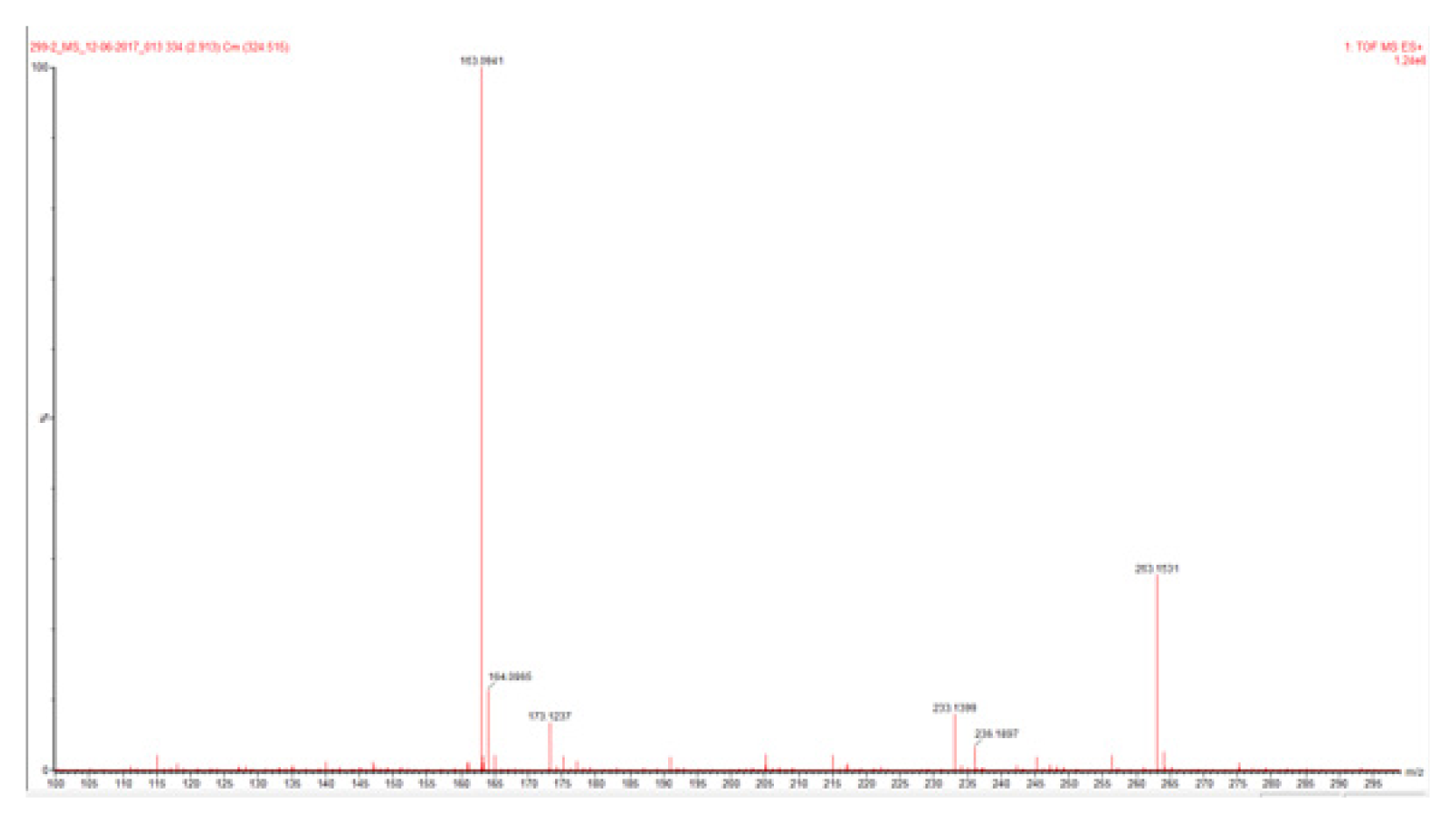

Supplement: Figure S10. — HR-ESI-MS of Compound 2. [file turkjchem-46-5-1468s10.tif]

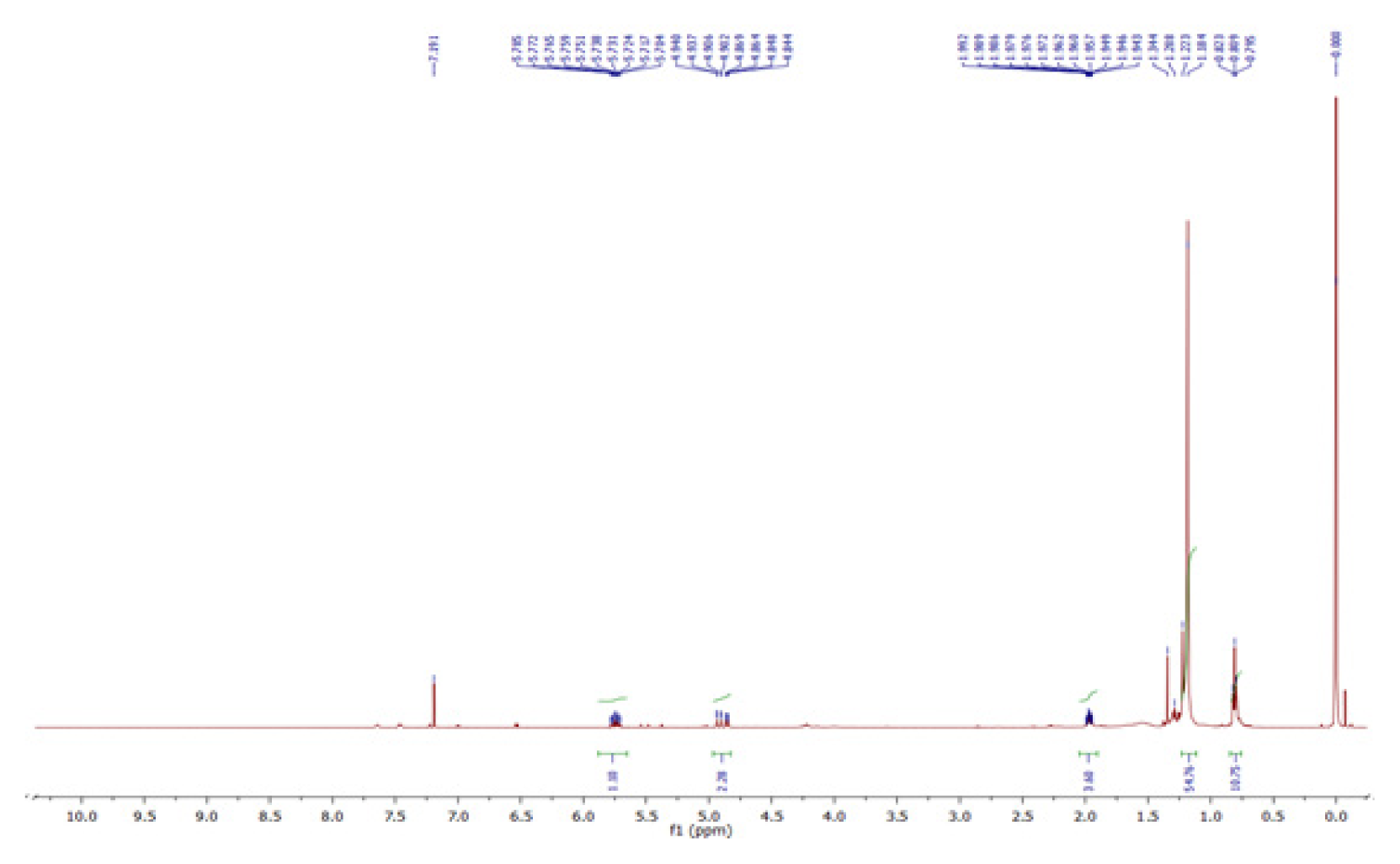

Supplement: Figure S11. — 1H NMR (CDCl3, 500MHz) Spectra of Compound 3. [file turkjchem-46-5-1468s11.tif]

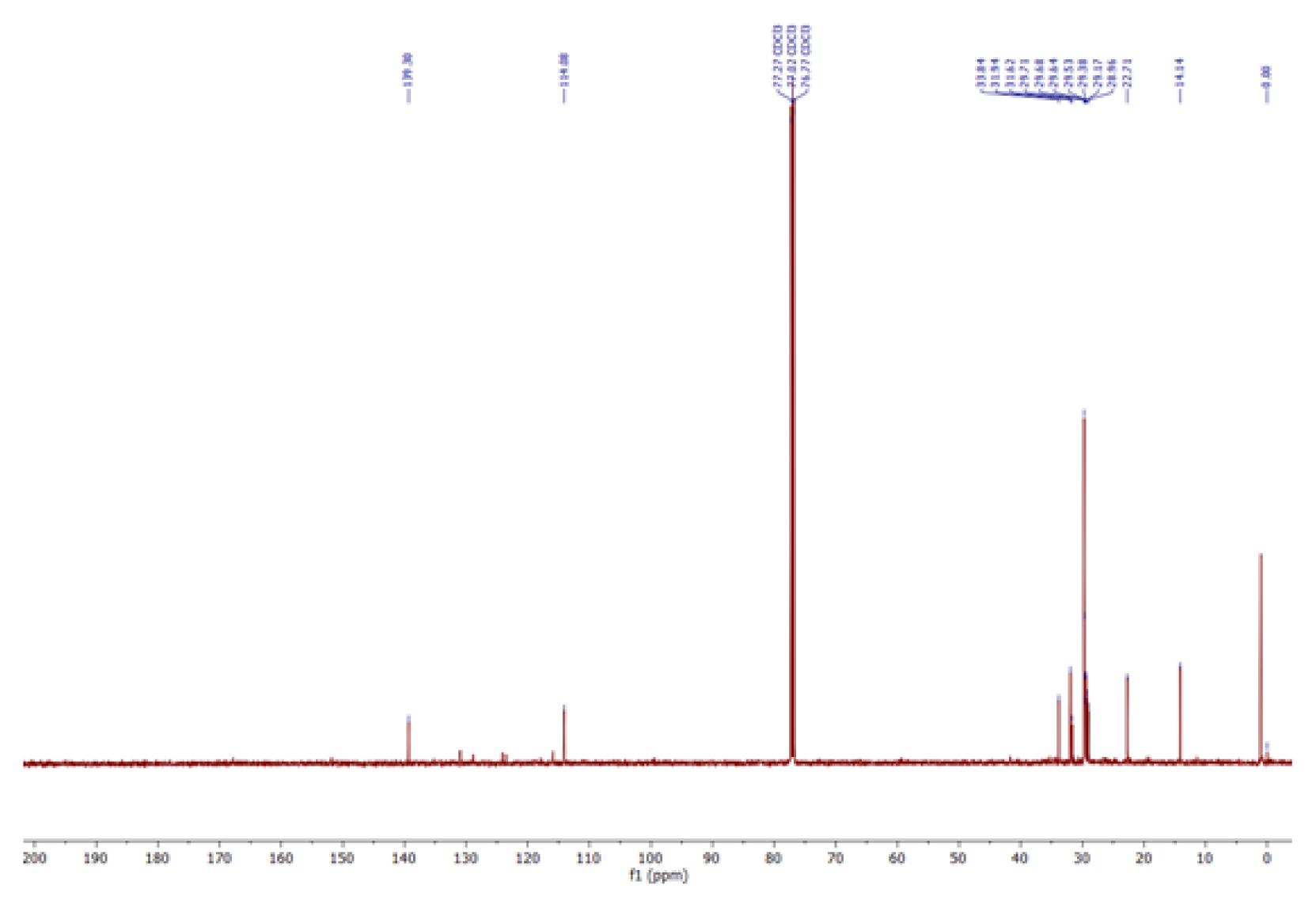

Supplement: Figure S12. — 13C NMR (CDCl3, 125MHz) Spectra of Compound 3. [file turkjchem-46-5-1468s12.tif]

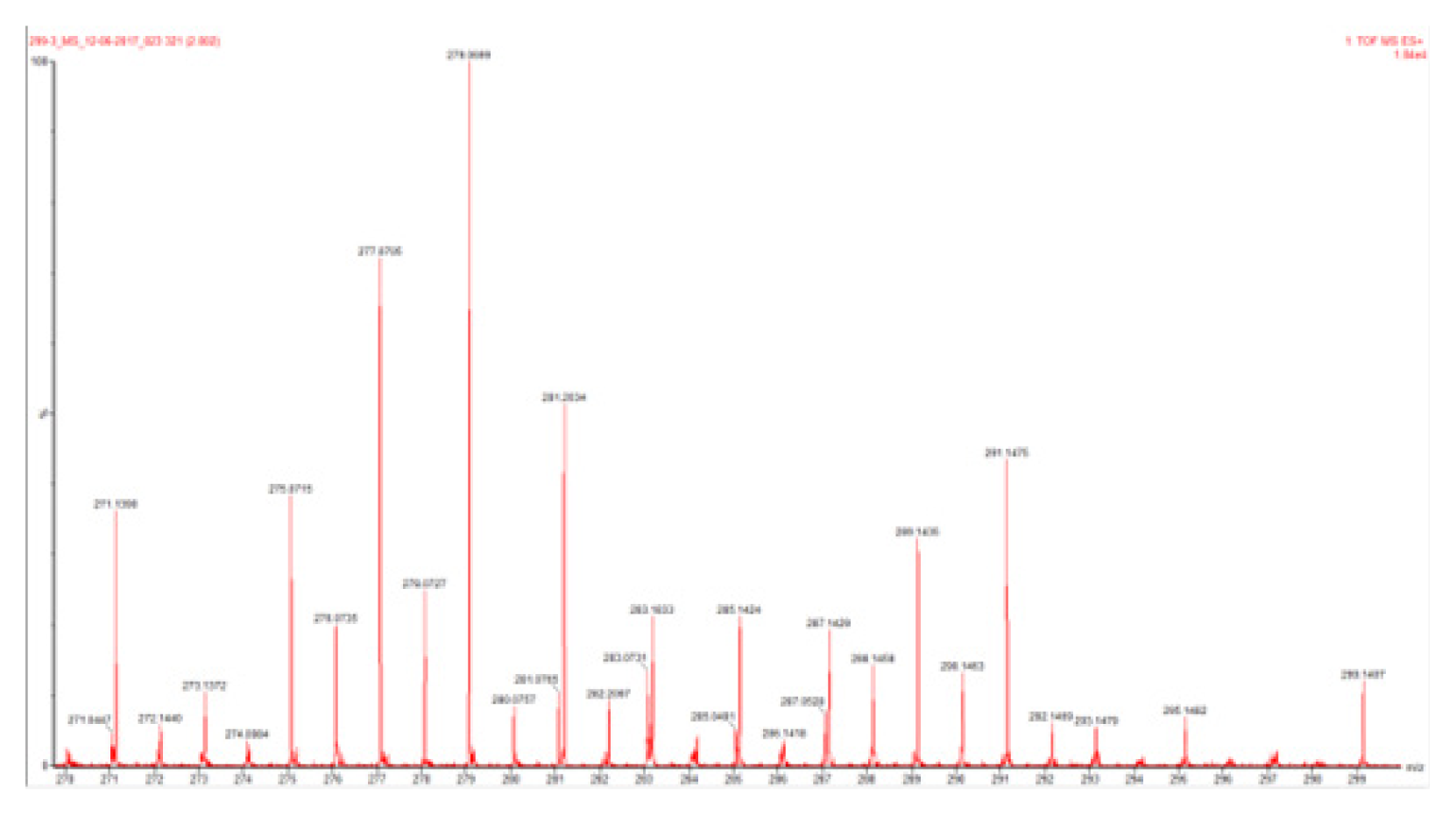

Supplement: Figure S13. — HR-ESI-MS of Compound 3. [file turkjchem-46-5-1468s13.tif]

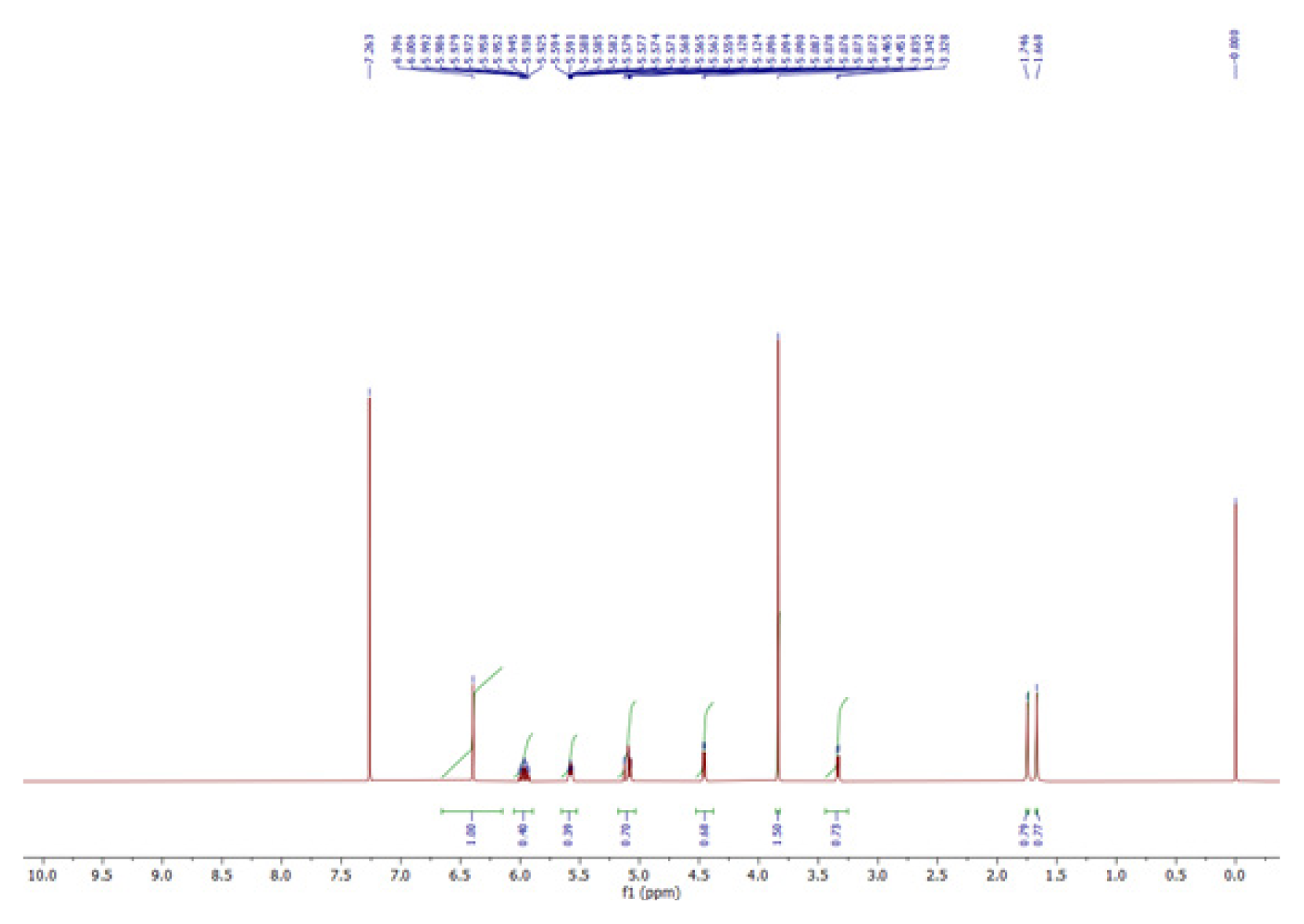

Supplement: Figure S14. — 1H NMR (CDCl3, 500MHz) Spectra of Compound 4. [file turkjchem-46-5-1468s14.tif]

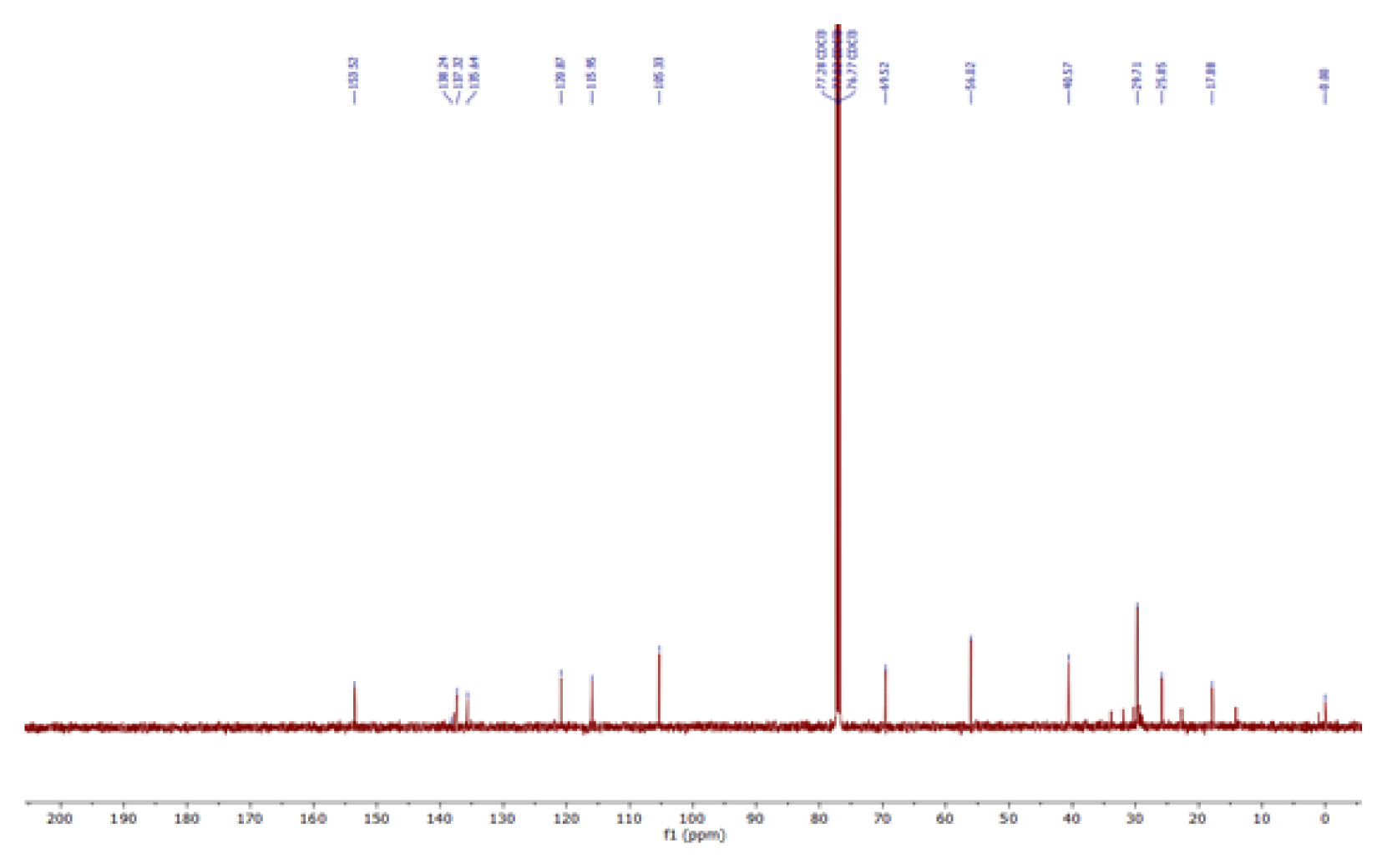

Supplement: Figure S15. — 13C NMR (CDCl3, 125MHz) Spectra of Compound 4. [file turkjchem-46-5-1468s15.tif]

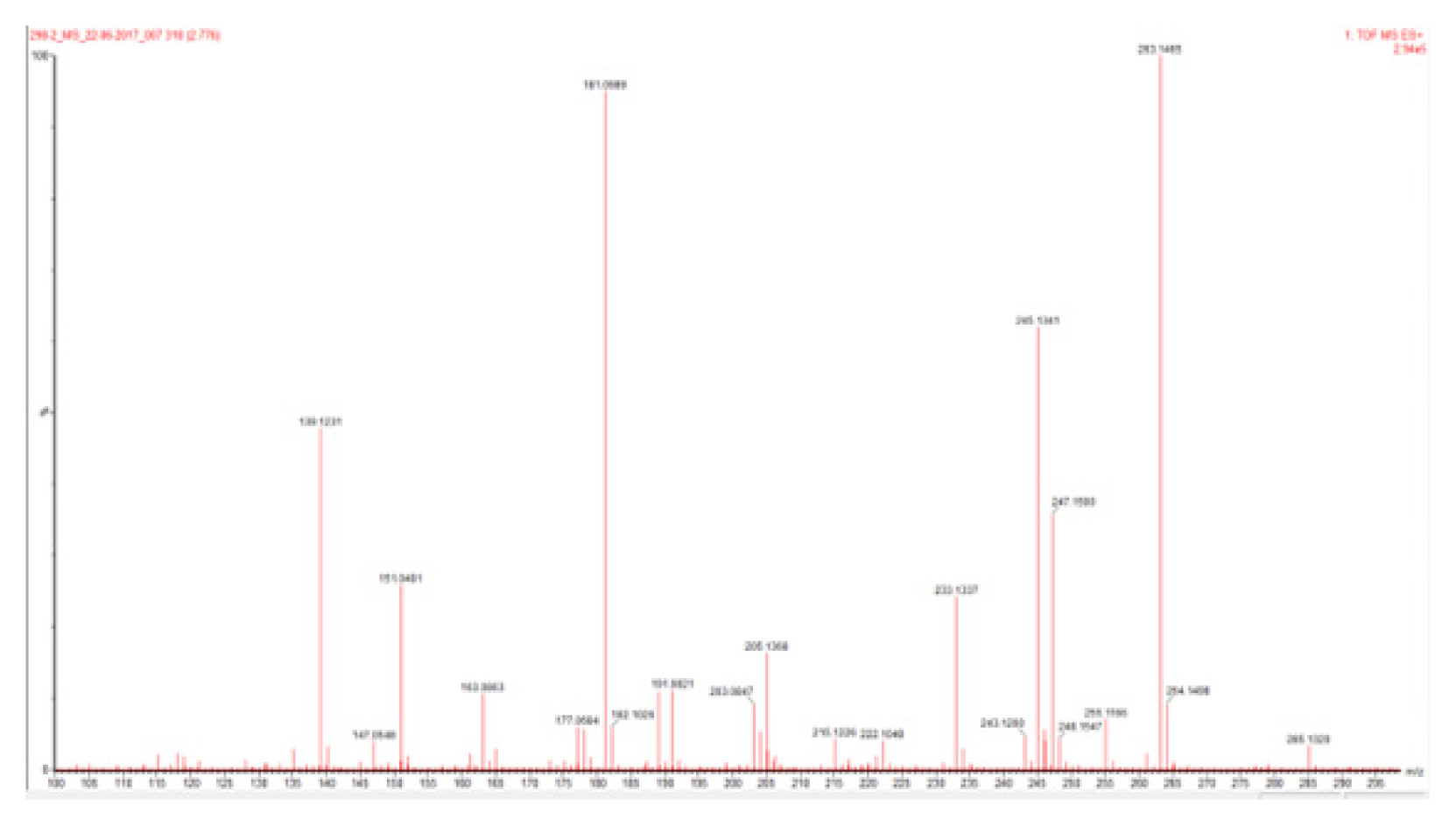

Supplement: Figure S16. — HR-ESI-MS of Compound 4. [file turkjchem-46-5-1468s16.tif]

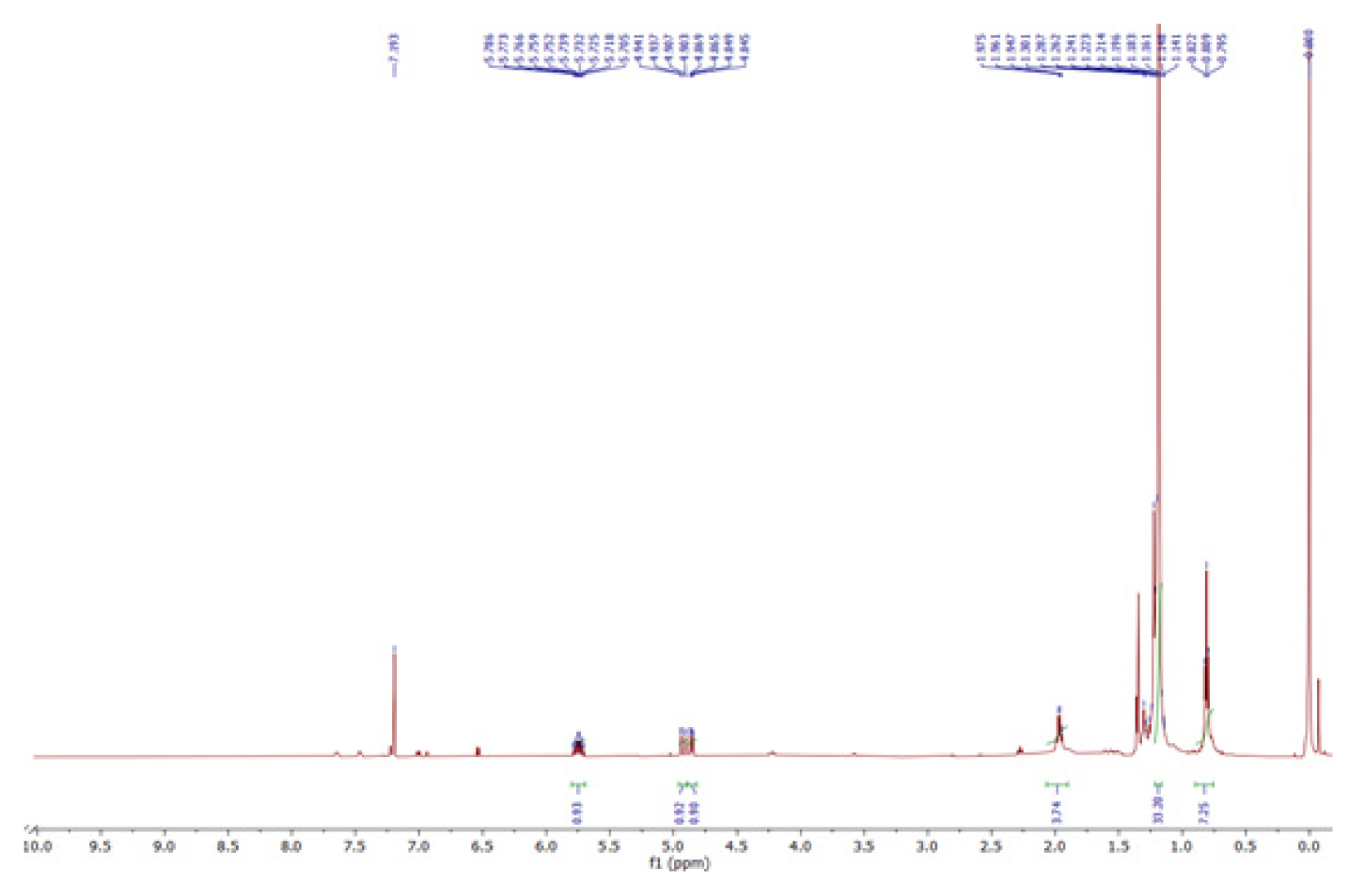

Supplement: Figure S17. — 1H NMR (CDCl3, 500MHz) Spectra of Compound 5. [file turkjchem-46-5-1468s17.tif]

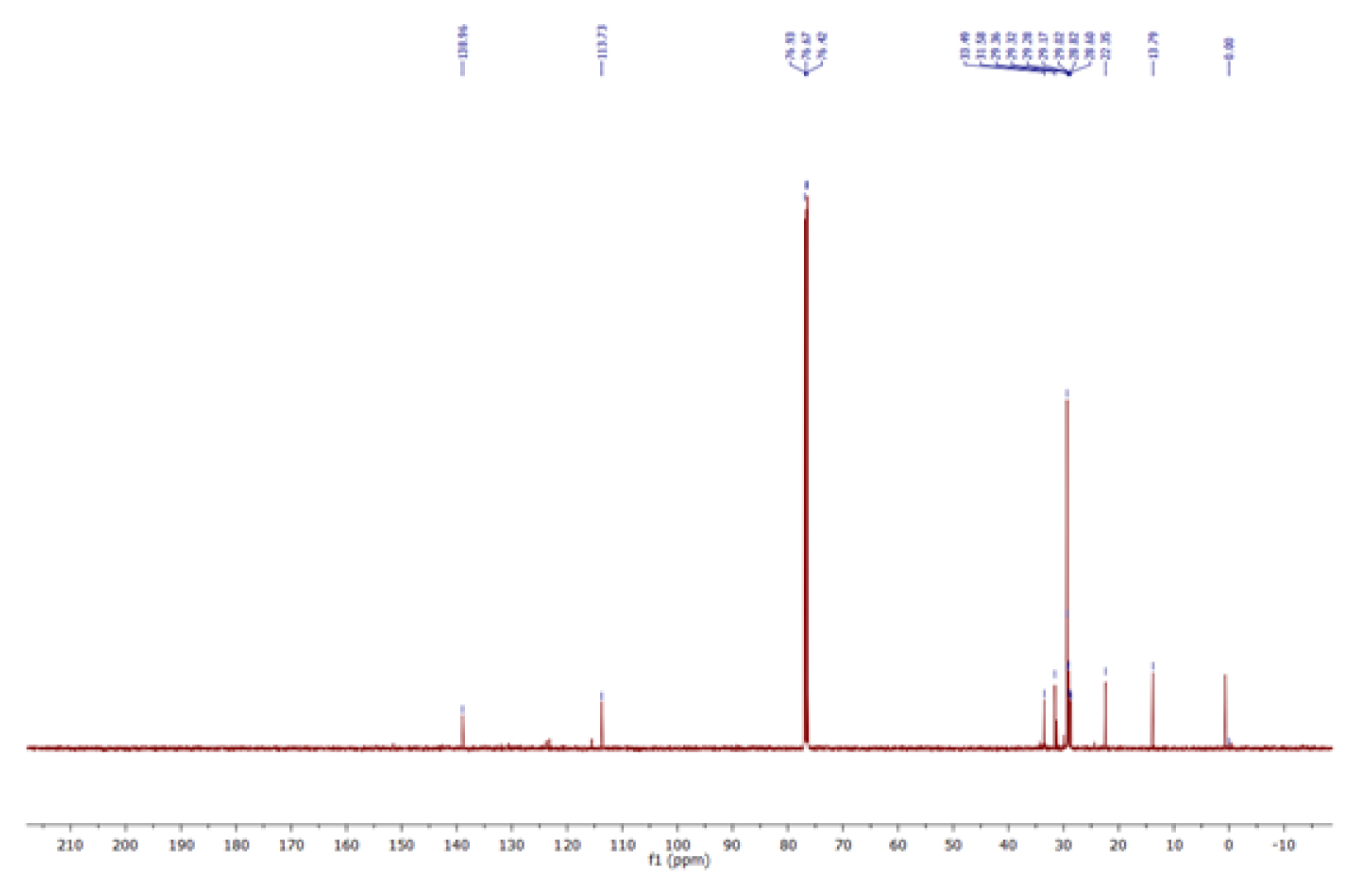

Supplement: Figure S18. — 13C NMR (CDCl3, 125MHz) Spectra of Compound 5. [file turkjchem-46-5-1468s18.tif]

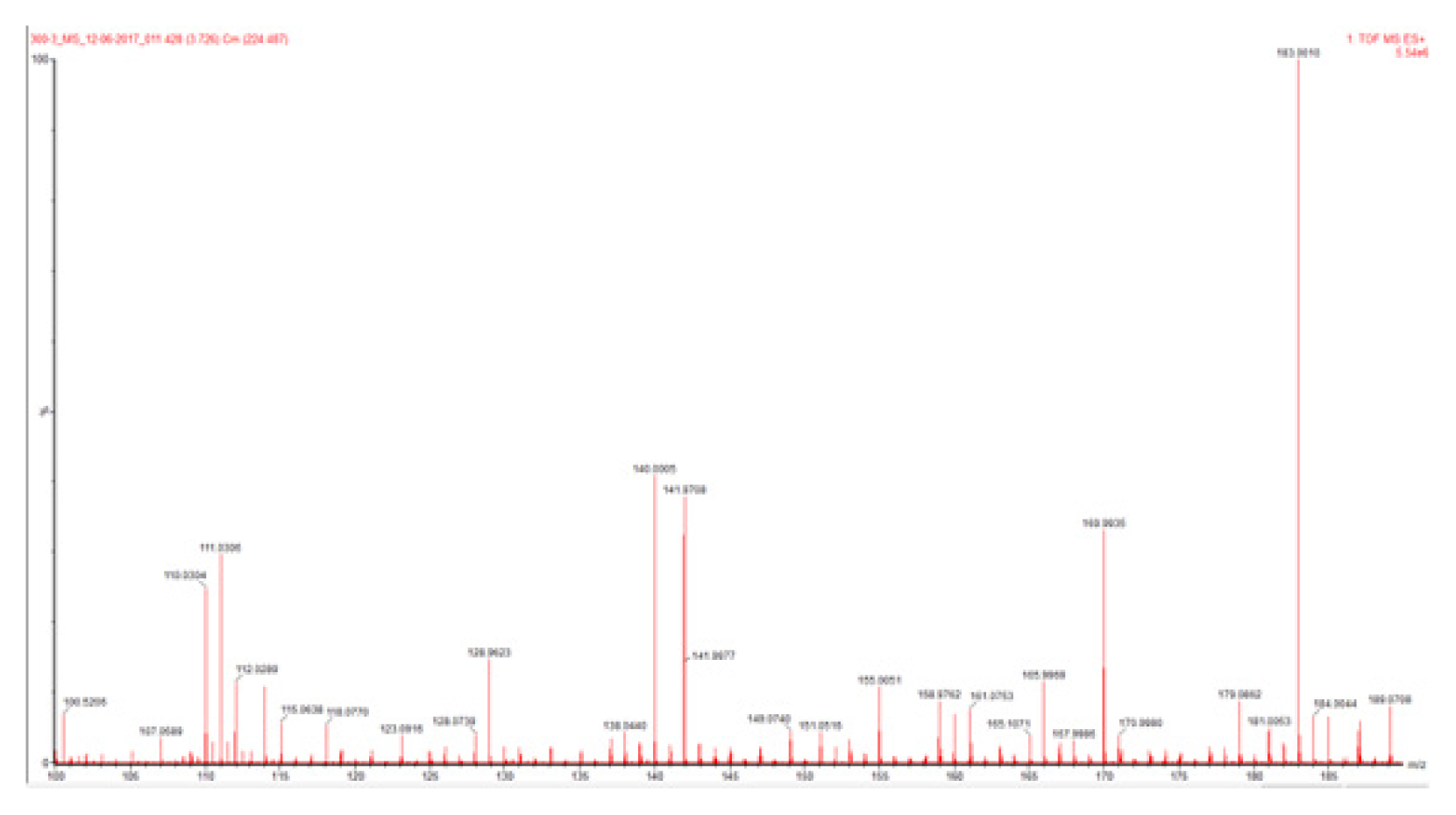

Supplement: Figure S19. — HR-ESI-MS of Compound 5. [file turkjchem-46-5-1468s19.tif]

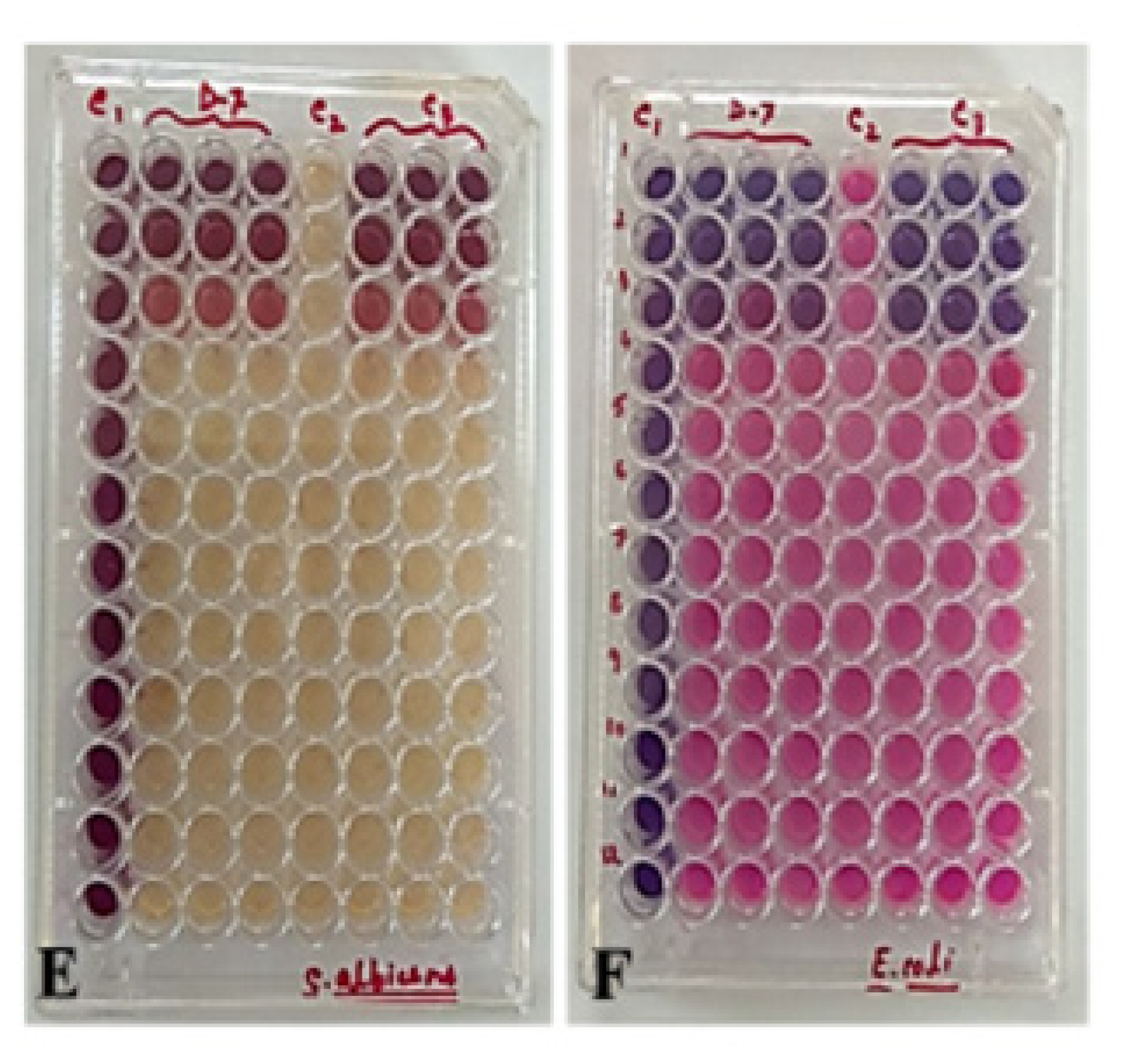

Supplement: Figure S20. — MIC of Compound 1 (E–F). [file turkjchem-46-5-1468s20.tif]
